# The Fungus *Candida albicans* Tolerates Ambiguity at Multiple Codons

**DOI:** 10.3389/fmicb.2016.00401

**Published:** 2016-03-31

**Authors:** João Simões, Ana R. Bezerra, Gabriela R. Moura, Hugo Araújo, Ivo Gut, Mónica Bayes, Manuel A. S. Santos

**Affiliations:** ^1^Health Sciences Program, Department of Medical Sciences, Institute of Biomedicine - iBiMED, University of AveiroAveiro, Portugal; ^2^Centro Nacional de Análises Genómico, Parc CientíficBarcelona, Spain

**Keywords:** *Candida albicans*, chimeric tRNA, stress, genome, evolution

## Abstract

The ascomycete *Candida albicans* is a normal resident of the gastrointestinal tract of humans and other warm-blooded animals. It occurs in a broad range of body sites and has high capacity to survive and proliferate in adverse environments with drastic changes in oxygen, carbon dioxide, pH, osmolarity, nutrients, and temperature. Its biology is unique due to flexible reassignment of the leucine CUG codon to serine and synthesis of statistical proteins. Under standard growth conditions, CUG sites incorporate leucine (3% of the times) and serine (97% of the times) on a proteome wide scale, but leucine incorporation fluctuates in response to environmental stressors and can be artificially increased up to 98%. In order to determine whether such flexibility also exists at other codons, we have constructed several serine tRNAs that decode various non-cognate codons. Expression of these tRNAs had minor effects on fitness, but growth of the mistranslating strains at different temperatures, in medium with different pH and nutrients composition was often enhanced relatively to the wild type (WT) strain, supporting our previous data on adaptive roles of CUG ambiguity in variable growth conditions. Parallel evolution of the recombinant strains (100 generations) followed by full genome resequencing identified various strain specific single nucleotide polymorphisms (SNP) and one SNP in the deneddylase (JAB1) gene in all strains. Since JAB1 is a subunit of the COP9 signalosome complex, which interacts with cullin (Cdc53p) to mediate degradation of a variety of cellular proteins, our data suggest that neddylation plays a key role in tolerance and adaptation to codon ambiguity in *C. albicans*.

## Introduction

Genetic code alterations have been reported in numerous organisms belonging to the three kingdoms of life. Sixteen of these changes were discovered in mitochondria and 10 in prokaryotes and eukaryotic nuclear codes (Knight et al., 2001; Di Giulio, 2005). The stop codons UAA, UAG, and UGA (Söll and RajBhandary, 2006) are the main targets of codon reassignment. In numerous green algae, ciliates and Diplomonads the UAA and UAG codons are used as glutamate (Sánchez-Silva et al., 2003). The UGA codon encodes tryptophan in *Mycoplasma* spp. (Yamao et al., 1985), *Spiroplasma citri* (Stamburski et al., 1992), *Bacillus* and various ciliates, and can also be used as a cysteine codon in Euplotes (Meyer et al., 1991). The mitochondrial UGA stop codon is normally used to incorporate tryptophan into proteins (Watanabe and Yokobori, 2011). The UAG codon is assigned to alanine (Ala) and leucine (Leu) in the mitochondria of fungi and several algae (Knight and Landweber, 2000; Kück et al., 2000; Söll and RajBhandary, 2006). In platyhelminth and echinoderm the lysine (Lys) AAA codon is assigned to asparagine (Castresana et al., 1998). Additionally the arginine codons AGA and AGG are used as Ser in most invertebrates and as stop codon in vertebrates, whereas tunicate (urochordata) mitochondria use AGA for glycine (Gly) (Watanabe and Yokobori, 2011). The serine UCA codon is also a stop codon in the green algae *Scenedesmus obliques* (Kück et al., 2000). Apart from this, selenocysteine (the 21st amino acid of the code) is incorporated into the active site of selenoproteins through UGA reprograming (Ambrogelly et al., 2007). Pyrrolysine, the 22nd amino acid, is incorporated into proteins in response to the UAG codon of *Methanosarcinaceae* (Srinivasan et al., 2002; Söll and RajBhandary, 2006).

Moreover, bacterial, fungal, and mammalian cells are tolerant to incorporation of artificial amino acids (Cropp and Schultz, 2004), supporting the codon assignment flexibility described above. For example, Wang and coworkers expressed an orthogonal tyrosyl-tRNA synthetase from *Methanococcus jannaschii* and a mutant Tyr amber suppressor tRNA in *Escherichia coli* (Wang et al., 2001). This pair incorporates the synthetic amino acid O-methyl-L-tyrosine into proteins in response to an amber nonsense codon (UAG). Since the orthogonality prevents the new tRNA-synthetase (aaRS) from aminoacylating cellular tRNAs and the recognition of the new recombinant tRNA by the host tRNA-synthetases, the system is highly specific (Wang et al., 2001). Anderson and co-workers were also able to engineer an orthogonal aaRS-tRNA pair derived from archaeal tRNA^Lys^ which efficiently and selectively incorporates a non-canonical amino acid into proteins in response to the quadruplet codon AGGA (Anderson et al., 2004). Genetic code engineering has also been achieved using an editing defective aaRS. Deletion of the editing domain of an isoleucyl-tRNA synthetase resulted in ambiguous translation of isoleucine (Ile) codons with non-canonical amino acids (Pezo et al., 2004). Finally, tRNAs bearing mutations in the anticodon, that do not affect aminoacylation, have been used to introduce various amino acids at non-cognate codons. For example Ser has been incorporated at 19 different codons in chick embryos and human cells (Geslain et al., 2010).

*Candida albicans* incorporates Ser at Leu CUG codons (Santos and Tuite, 1995), using a unique ${\text{tRNA}}_{\text{CAG}}\text{Ser}$ that contains a leucine (5′-CAG-3′) anticodon and is aminoacylated by the seryl-synthetase (serRS). Interestingly, anticodon mutations (A35 and m^7^G_37_) are also recognized by the leucyl-tRNA synthetase (LeuRS) and the ${\text{tRNA}}_{\text{CAG}}\text{Ser}$ is also charged with Leu at low level (Soma et al., 1996), resulting in two distinctly charged aminoacyl-tRNAs in *C. albicans* cells: the ${\text{Ser-tRNA}}_{\text{CAG}}\text{Ser}$ and ${\text{Leu-tRNA}}_{\text{CAG}}\text{Ser}$. Since ${\text{Leu-tRNA}}_{\text{CAG}}\text{Ser}$ is not edited by the LeuRS nor discriminated by the translation elongation factor 1 (eEF1A; Santos et al., 1997, 2011), it participates in protein synthesis, creating CUG ambiguity (Gomes et al., 2007). Under standard growth conditions 3% of Leu and 97% Ser are incorporated at CUG sites on a proteome wide scale (Gomes et al., 2007). Remarkably, incorporation by Leu at CUG sites can be artificially increased up to 98% (Bezerra et al., 2013). To determine if such flexibility is specific of the CUG codon or also exists in other codons, we have introduced various anticodons in a tRNA^Ser^ to misincorporate Ser at multiple non-cognate codon sites. There was small decrease in fitness, but the recombinant strains recovered growth rate relatively fast, indicating that *C. albicans* tolerance to codon ambiguity is widespread. Phenotypic profiling testing growth in variable conditions, namely temperature, pH, antifungal drugs and nutritional cues, showed advantageous growth of the ambiguous strains relative to the control strain. Protein aggregation was observed in the strain that misincorporated Ser at the Leu CTC codon and increased DNA content was detected in strains misreading the other Leu codons (CTA and CTC). The effect of the misreading tRNAs in genome evolution was also investigated. After 100 generations, strains misreading Leu CTC and Ala GCC codons showed loss of heterozygosity (LOH) in chromosomes II, III, and R and there was rapid accumulation of single nucleotide polymorphisms (SNP). These results are in line and support previous studies from our laboratory on the phenotypic and genomic effects of increased CUG ambiguity in *C. albicans* (Bezerra et al., 2013).

## Materials and methods

### Strains and growth conditions

*E. coli* strain JM109 (*recA*1, *endA*1, *gyrA*96, *thi, hsdR*17, *supE*44, *relA*1, Δ(*lac-proAB*)/F′ [*traD*36, *proAB*^+^, *lacI*^q^, *lacZ*ΔM15]) was used in all DNA manipulations.

*C. albicans* strain SN148 (arg4Δ/arg4Δ leu2Δ/leu2Δ his1Δ/his1Δ ura3Δ::imm^434^/ura3Δ::imm^434^ iro1Δ::imm^434^/ iro1Δ::imm^434^) (Noble and Johnson, 2005) was grown in YPD (1% yeast extract, 2% peptone, 2% dextrose) at 30°C. Transformed strains were grown in synthetic minimal medium (0.67% yeast nitrogen base, 2% glucose, 0.2% Drop-out mix with all the essential amino acids).

### Plasmid purification and construction

The plasmids used in this study were purified using GeneJet^TM^ Plasmid Miniprep Kit (Fermentas) according to the manufacturer's instructions. Plasmid pUA552 was constructed by PCR amplification of tDNAser (UGA) from *S. cerevisiae*, with oligo oUA1671 5′-AAAGGTACCGAAGGA GGTGCAAGGGAAAAG-3′ and oUA1672 5′-TTTGGG CCCTCCGTGCATAACGAATGACTC-3′ and inserted between *KpnI* and *ApaI* restriction enzyme sites of PMG2287 (gift from Prof. Judith Berman). The tRNA was mutated using the QuikChange Site-Directed Mutagenesis kit (Stratagene) with oligos oUA1766 5′-TAAGGCGACAGACGT GAAATCTGTTGGGCTC-3′ and oUA1767 5′-GAGCCCAAC AGATTTCACGTCTGTCGCCTTA-3′ with the purpose of exchanging T33 for G33 to decrease serylation efficiency of the tRNA (Santos et al., 1996). Then plasmid pUA552 (tRNA anticodon) was mutated using the oligonucleotides described in Supplementary Table 1 to produce new plasmids. These plasmids were linearized with *StuI* and integrated into the *C. albicans* genome.

### *C. albicans* transformation

Transformation of *C. albicans* was carried out using an improved lithium acetate method (Walther and Wendland, 2003) with small modifications. Briefly, overnight cultures were inoculated in fresh medium to an O.D_600nm_ of 0.3 and allowed to grow at 30°C, 200 rpm with shaking until an O.D_600nm_ of 1–1.2. Cell cultures were then transferred to 50 ml falcon tubes and centrifuged at 3000 rcf for 5 min, supernatants were discarded and pellets were resuspended in 1.5 ml of LiAc-solution (0.1 M LiAc, TE buffer 1x). Two-hundred microliters of suspension were transferred to 1.5 mL eppendorf tubes and the transformation reagents were added in the following order: 600 μL 50% (w/w) PEG LiAc-solution, 50 μL single-stranded carrier DNA (2 mg/mL) previously denatured and 50 μL of an aqueous solution of the plasmid of interest (containing 1–5 μg of plasmid). Tubes were vortexed and then incubated at 30°C for 4 h. Followed by an incubation at 44°C for 15 min and then placed on ice for 2 min. Cells were harvested at 4500 rcf for 5 min and resuspended gently in 200 μl of appropriate SC selective medium. Each 100 μl of suspension was plated onto appropriate SC selection medium plates and incubated at 30°C for 3–4 days.

### Plasmid integration

Correct plasmid integration at the *RPS10* locus was confirmed by PCR using the primers oUA1554 5′-CGTAT TCACTTAATCCCACAC-3′, oUA1555 5′-CCAATT GGTGATGGTCC-3′. Single copy integration was selected using the primers oUA1556 5′-GGTATAGAAATGCTGGTTGG-3′ and oUA1557 5′-CCAATTGGTGATGGTCC-3′, which produced a single PCR amplification product for tandem integrated plasmids (Barelle et al., 2004).

### Northern blot analysis

About 200 mL of exponentially growing cells were harvested by centrifugation at 3000 rcf for 4 min at room temperature. After removal of the supernatant, tubes were immediately immersed in liquid nitrogen for 6 min, frozen and stored at −80°C. Total RNA was isolated from yeast cells using a hot phenol based protocol (Schmitt et al., 1990). Frozen pellets were resuspended in 500 μl acidic phenol-chlorophorm mixture (Sigma, 5:1, pH 4.7) at 65°C. An equal volume of TES-buffer (10 mM Tris pH 7.5, 10 mM EDTA and 0.5% sodium dodecyl sulfate) was added and each tube was vortexed at high speed for 20 s, to resuspend pellets. Tubes were then incubated for 1 h in a water bath at 65°C with 20 s vortexing every 10 min. Tube's content was transferred to 1.5 ml tubes and centrifuged for 20 min at 16000 rcf at 4°C. The water-phase was added to a new tube, filled with 600 μl Acid Phenol Chloroform (Sigma, 5:1, pH 4.7) vortexed for 20 s and centrifuged for 10 min at 16000 g, at 4°C. The previous step was repeated two more times with 500 and 400 μl Acid Phenol Chloroform (Sigma, 5:1, pH 4.7), respectively. Finally, the water-phase was added to new microfuge tubes with 35 μl sodium acetate (3 M, pH 5.2), 800 μl ethanol (100%, kept at −20°C), and incubated at −20°C for at least 1 h. Tubes were then centrifuged for 5 min at room temperature, 16000 g, supernatants were removed carefully with a pipette tip, avoiding touching the RNA-pellet. Pellets were then washed with 500 μl ethanol (80%, −20°C) and centrifuged for 3 min at room temperature, 16000 g. Ethanol was removed and the RNA pellets were air dried for 1 min and dissolved in sterile water to a concentration of 10 μg/μl. Samples were stored at −80°C. Fifty micrograms of total RNA were resolved at room temperature in 15% polyacrylamide (40% Acril:Bis) gels containing 8 M urea, buffered with 1X TBE pH 8.0, electrophoresed at 500 V for 16 h. The section of the gels containing the tRNAs was transferred to a nitrocellulose membrane (Hybond N, Amershan) by a Semi-Dry Trans Blotting system (Bio-Rad). Probes 5′-ACGCTCTACCACTAAGCTAA-3′ for detection of ${\text{tRNA}}_{\text{UGC}}\text{Thr}$ and 5′-TTAACCACTCGGCCATAGT-3′ for detection of ${\text{tRNA}}_{\text{UGA}}\text{Ser}$, were prepared by phosphorylating 10 pmol of dephosphorylated oligonucleotide with 4 μl of ɤ-^32^P-ATP (5000 Ci/mmol) (Perkin Elmer) in 1X T4 Kinase buffer, 10 mM spermidine and 16 units of T4 Kinase (Takara). This reaction was carried out by incubation for 1 h, at 37°C. Probes were purified with 100 μl phenol:chlorophorm:isoamyl alcohol (PCIA) and hybridized in a hybridization solution [6x SSPE (20x SSPE: 3 M sodium chloride, 0.2 M sodium phosphate, 0.02 M Na_2_EDTA), 5x Denhardt's solution (50x Denhardt's solution = 0.02% bovine, serum albumin, 0.02% polyvinylpyrrolidone and 0.02% Ficoll), 0.05% sodium dodecyl sulfate], overnight at 52°C. Hybond-N membranes were washed with washing solution (2X SSPE, 0.5% sodium dodecyl sulfate), wrapped in a plastic bag and exposed for 24 h to a K-screen and scanned using Molecular Imager FX (Bio-Rad) with adequate settings.

### Protein synthesis

Quantification of protein synthesis was performed as described before with few alterations (Alamgir et al., 2008). Briefly, 2 × 10^8^ mid exponential growing phase cells were collected and washed three times at 30°C with minimal medium lacking Met and Uri. Cells were then resuspended in 2 ml of the same pre-warmed minimal medium and incubated for 20 min at 30°C with agitation. Two microliters of cold L-[^35^S]-Methionine (Perkin Elmer, 1175 Ci/mmol, 10.5 mCi/ml) were added and the mixture was incubated for 8 min at 30°C, with 200 rpm of agitation. Amino acid incorporation was stopped by addition of 60 μl of cycloheximide (20 mg/ml) and incubation on ice. Cells were washed three times with cold water and frozen at −80°C. Total protein was then extracted by resuspending cell pellets in 200 μl Lysis buffer [50 mM potassium phosphate buffer pH 7, 1 mM EDTA, 5% (vol/vol) glycerol, 1 mM phenylmethylsulfonyl fluoride, and complete mini protease inhibitor cocktail (Roche)] and 120 μl of glass beads. Cells were disrupted using a Precellys disrupter (5 cycles of 10 s at 5000 rpm and 1 min on ice between cycles) and centrifuged at 3000 rcf for 10 min. Thirty microliters of supernatant were applied onto 1 cm^2^ square paper microfiber filters (GF/C, Whatman). Radioactivity counting was performed using a scintillation counter (Beckman). Protein extracts were quantified using the BCA protein quantification Kit (Pierce). Counting was normalized against the total protein for each sample and compared to control.

### Phenotypic assays

Phenotypic assays were performed as previously described (Homann et al., 2009) with minor alterations. Briefly, 1 × 10^8^ cells from mid exponential phase cultures were collected and resuspended in 1 ml of water. Six 10x dilutions were transferred to 96-well micro plates. This format allowed for cell plating using a liquid handling station (Caliper LifeSciences). The assay plates (agar) were incubated for 5 days and colonies were photographed using a dissecting microscope equipped with AxioCam HRc camera and Axio Vision Software from Zeiss. Images were analyzed using Image J and colonies size was determined. Growth scores (GS) of each strain were calculated by dividing the size of the colonies in the assay plate by the size of the colonies in the control plates, for the corresponding dilutions (Supplementary Equations 1, 2). From these growth scores of mistranslating strains (GS_m_) the growth score of control strain (GS_c_) was subtracted, producing a relative growth score (RGS) for each mistranslating strain in each assay. Assay plates conditions are described in (Supplementary Table 2).

### Insoluble protein quantification

Quantification of insoluble protein was carried out as described previously (Rand and Grant, 2006), with minor modifications. Cells were grown to exponential phase in MM-Uri. Equal number of cells were harvested by centrifugation, washed and transferred to 2 ml microtubes and frozen at −80°C for 30 min. Tubes were removed from the freezer and 300 μl of lysis buffer [50 mM potassium phosphate buffer, pH 7, 1 mM EDTA, 5% (vol/vol) glycerol, 1 mM phenylmethylsulfonyl fluoride (PMSF), and complete Mini protease inhibitor cocktail (Roche)] plus lyticase (20 mg/ml) were added to each tube. Reaction mixtures were incubated for 30 min at 37°C. Cells were disrupted with 100 μl of glass beads using a Precellys 24 (bertin technologies), three cycles at 5000 rpm for 10 s, followed by 2 min of incubation on ice. Mixtures were then centrifuged at 3000 rcf for 15 min at 4°C, to remove intact cells, and supernatants were transferred to new tubes. Another centrifugation was carried out at 15000 rcf for 20 min to isolate membranes and protein aggregates. Next, membrane proteins were removed by washing pellets twice with 320 μl of lysis buffer complemented with 80 μl of 10% Triton-X100, centrifuging at 15000 rcf for 20 min each time. Finally aggregated proteins were resuspended in 50 μl of lyses buffer and 10 μl of 6 X sample buffer (30% glycerol, 10% sodium dodecyl sulfate, 0.6 M DTT, and 0.012% bromophenol blue in 0.5 M Tris-Cl/0.4% SDS, pH 6.8) and boiled for 5 min at 95°C. Aliquots of 10 μl of each sample were fractionated on 15% acrylamide gels. Following electrophoresis, gels were stained by submersion on a coomassie blue solution (0.25% Brilliant Blue R, 50% methanol, and 10% acetic acid) for 2 h with slow agitation. Destaining was carried out using 25% methanol and 5% acetic acid solutions, overnight with slow agitation. Gels were then washed with distilled water and scanned using the ODYSSEY Infrared Imaging System (Li-Cor Biosciences) with the adequate definitions. Gel images obtained were analyzed with ImageJ software and gel bands intensity was quantified.

### Evolution experiments

Strains were grown in rich medium until stationary phase and 0.5% of each culture was then inoculated in fresh rich medium. Evolution was carried out for ~100 generations.

### DNA extraction

Genomic DNA extraction was carried out using the Genomic-tip 100/G kit (Qiagen) according to the manufacturer's guide. DNA quantification and quality was carried out using Picogreen fluorescence based quantification assay.

### Illumina whole-genome sequencing and data analysis

Genomic DNA was sequenced using the Illumina HighSeq sequencers, using the manufacturer-supplied protocols and reagents. One library was prepared using Illumina DNA Sample preparation protocol with an insert size of 400–500 bp. Succinctly, 5 μg of high-molecular weight genomic DNA was fragmented by a Covaris sonication device and DNA fragments were end-repaired and A-tailed. Adapters were ligated by a 3′ thymine overhang and amplified by PCR. The library was applied to an Illumina flow cell for cluster generation and sequencing was carried out. Raw sequencing data (146-bp paired-end reads with expected insert size of 400–500 bp) from each sample were trimmed by removing consecutive bases on 5′ and 3′ flanks with base quality less than 20. Trimmed reads that did not pass filtering criteria for ambiguity (N content < 5%), complexity (score ≥ 10), length (50 bases or longer), and average base quality ≥20 were removed using Bamtools (Barnett et al., 2011). Remaining reads were mapped to the reference genome of *C. albicans* obtained from the Candida genome database (http://www.candidagenome.org/, assembly 21) using BWA (Li and Durbin, 2010). Processing and filtering of mapped reads were done using Samtools (Li et al., 2009). After removal of duplicates, read pairs aligning to opposite strands, or where predicted insert size not matching actual size were removed. In addition, read pairs were removed where one or both reads had low mapping quality (MQ < 20) or less than 95% sequence identity to the reference. Samtools was used to produce read pileups (Li et al., 2009), detect single nucleotide variants and call genotypes. Small insertions and deletions (indels) were not called. Bases with low base quality or read depth less than three or higher than twice the sample average coverage were called as unknown genotype. Sequencing data were archived in the European Nucleotide Archive under accession number PRJEB11905.

### Accession numbers

The accession numbers for genes mentioned in this paper are from Candida genome database (http://www.candidagenome.org/, assembly 21).

## Results

### Construction and expression of mutant misreading tRNAs

We have previously engineered codon misreading in *Sacharomyces serevisiae* and *C. albicans* strains, using a tRNASer construct that misincorporates Ser at the Leu CUG codon (Gomes et al., 2007; Paredes et al., 2012; Bezerra et al., 2013). Mass-spectrometry analysis of a target protein produced by these *S. cerevisiae* recombinant strains showed misincorporation levels in the 1.4 to 2.31% range (Silva et al., 2007). In the present study, we used the same strategy and mutated the anticodon of the yeast ${\text{tRNA}}_{\text{UGA}}\text{Ser}$ gene to produce anticodons for various non-cognate codons, namely for Leu (CTA, CTC, CTT), Ile (ATC), Ala (GCC), Gly (GGA), Lys (AAG), Thn (ACC), and Tyr (TAC) codons. Because the eukaryotic seryl-tRNA synthetase (SerRS) does not recognize the tRNA^ser^ anticodon (it binds to the acceptor stem, D-arm and extra stem/loop), the alterations in the anticodon of the ${\text{tRNA}}_{\text{UGA}}\text{Ser}$ do not alter charging specificity (Lenhard et al., 1999) and allow for partial decoding of those targeted codons as serine. Amino acids chemical properties were considered during planning of the tRNA anticodon site directed mutagenesis (Supplementary Table 3). Leu and Ile were chosen based on differences in molecular volume, molecular weight and hydropathy. Thr, Gly and Ala were selected due to similarity of molecular volume with Ser, and Tyr was selected for being an aromatic amino acid. Finally, Lys was chosen due to its polar basic amino acid group. Expression of these recombinant tRNAs in *C. albicans* was monitored by northern blot analysis. The only tRNA that we could not detect was the one that decoded the leucine CTC codon (Supplementary Figure 1), however the strain that expressed it showed the lowest transformation efficiency (Supplementary Figure 2) and growth rate (Supplementary Figure 3), suggesting that it was highly toxic even in very low abundance. This result is in line with previous yeast data where the expression of mistranslating tRNAs was strongly down regulated through unknown mechanisms (Silva et al., 2007; Paredes et al., 2012).

### Phenotypic and cellular consequences of the misreading tRNAs

To evaluate the impact of expression of the misreading tRNAs on *C. albicans* fitness, growth rate was determined. There was a negative effect on growth in all strains. Leu (CTC) and Thr (ACC) strains showed ~50 and 60% decrease in growth rate relative to the control pUA552, respectively. The strains misreading the Leu (CTA, CTT), Ile (ATC), Ala (GCC), and Gly (GGA) codons had 39, 25, 29, 46, and 34% decrease in growth rate, respectively (Supplementary Figure 3). These results are in agreement with previous studies showing that *S. cerevisiae* growth rate is affected by misreading tRNAs (Santos et al., 1996). Since, mistranslation induces proteotoxic stress and a recent yeast inducible mistranslation study showed down regulation of protein synthesis rate (Paredes et al., 2012), we decided to clarify if the different mutant tRNAs engineered in this study could also affect protein synthesis rate in *C. albicans*. For this, we have quantified the incorporation of [^35^S]-methionine into proteins *in vivo* using the pulse labeling method. Protein labeling was carried out by adding of 1 μCi of [^35^S]-methionine to actively growing cells (10^7^ cells). Protein synthesis was stopped by addition of cycloheximide and cells were crushed on ice. Protein synthesis rate decreased in all strains (Supplementary Figure 4), in particular in those that misread the Leu (CTC, CTT), Ala (GCC), and Lys (AAG) codons. In most cases, growth and protein synthesis rates were correlated, except in the strains mistranslating Thr codons.

**Figure 1:**
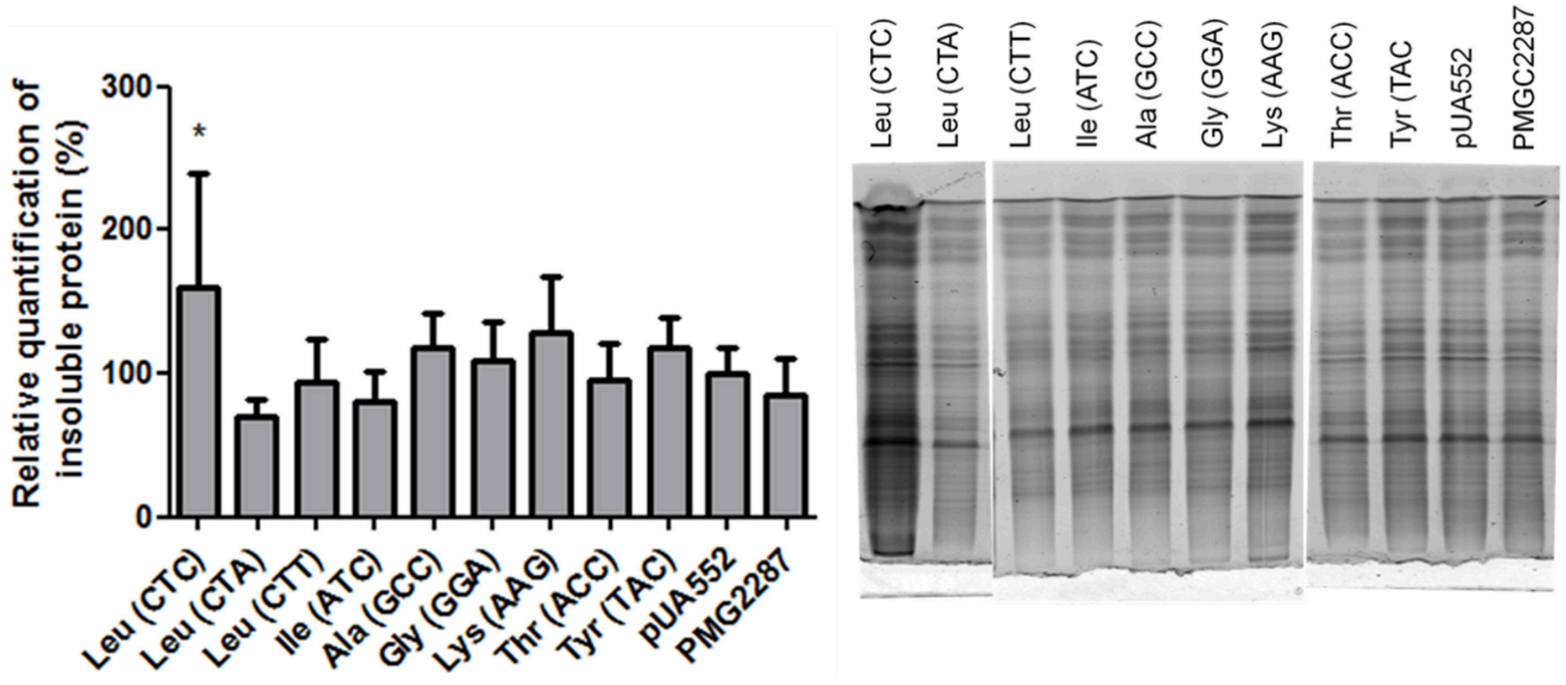
**The effect of mistranslation on protein aggregation**. The graph shows small increases in protein aggregation induced by mistranslation. The data represents insoluble protein + s.d. of duplicates of 3 different clones, normalized to the pUA552 control. Statistical analysis was carried out using one-way ANOVA was followed by a Dunnet test with CI 95% relative to pUA552 (^*^p < 0.05). Gel showing insoluble protein fractionated on a 15% acrylamide gel.

Several studies associate mistranslation with protein aggregation. A mouse mutation in the editing domain of the alanyl-tRNA synthetase results in misacylated Ser-tRNA^Ala^ and widespread translational errors, leading to the production of misfolded proteins, formation of large protein aggregates and death of Purkinje cells in the mouse cerebellum (Lee et al., 2006). In order to clarify if the *C. albicans* mistranslating strains also accumulated insoluble proteins the presence of aggregates was checked, but there was no significant difference between the control and mistranslating strains with the exception of the strain misincorporating Ser at the Leu CTC codon (Figure 1). The reason for this difference is not clear, but the CTC codon is used at low level and possibly the relative ratio of Ser misincorporation at this codon is higher than in the other cases, leading to production of higher level of erroneous proteins.

**Figure 2:**
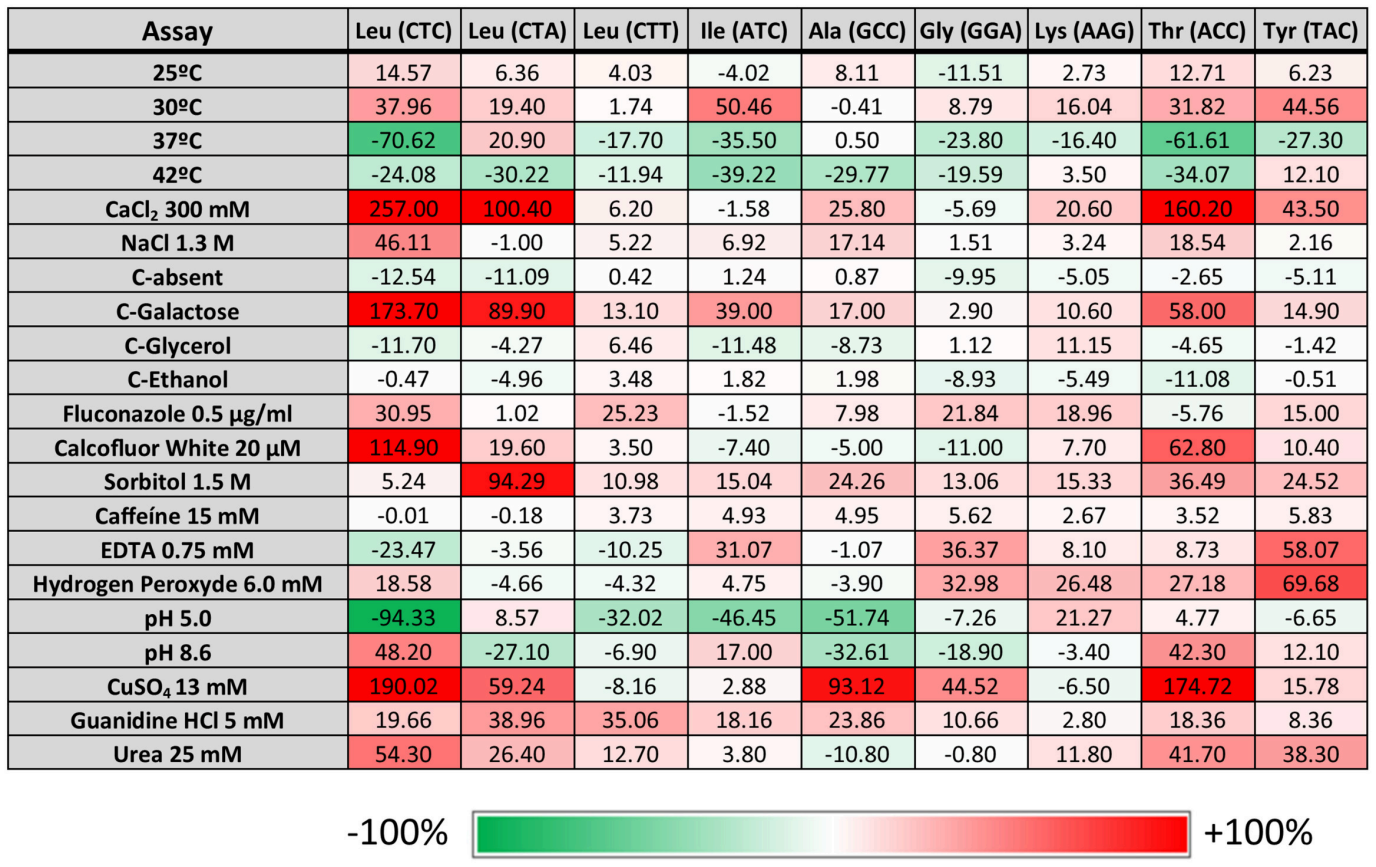
**Phenotypic assays**. Data represent the average size of the colonies obtained for all the transformed cells after 5 days of growth under the indicated conditions normalized with growth at 30°C and compared with the normalized control pUA552 (considered 0%) for that condition (relative growth score). The assay was performed with two independent growth cultures and three independent clones. Brick color intensity represents the strength of the phenotype. White squares represent a phenotype that is indistinguishable from the control strain pUA552. Green and red squares represent either a growth reduction or enhancement, respectively.

**Figure 3:**
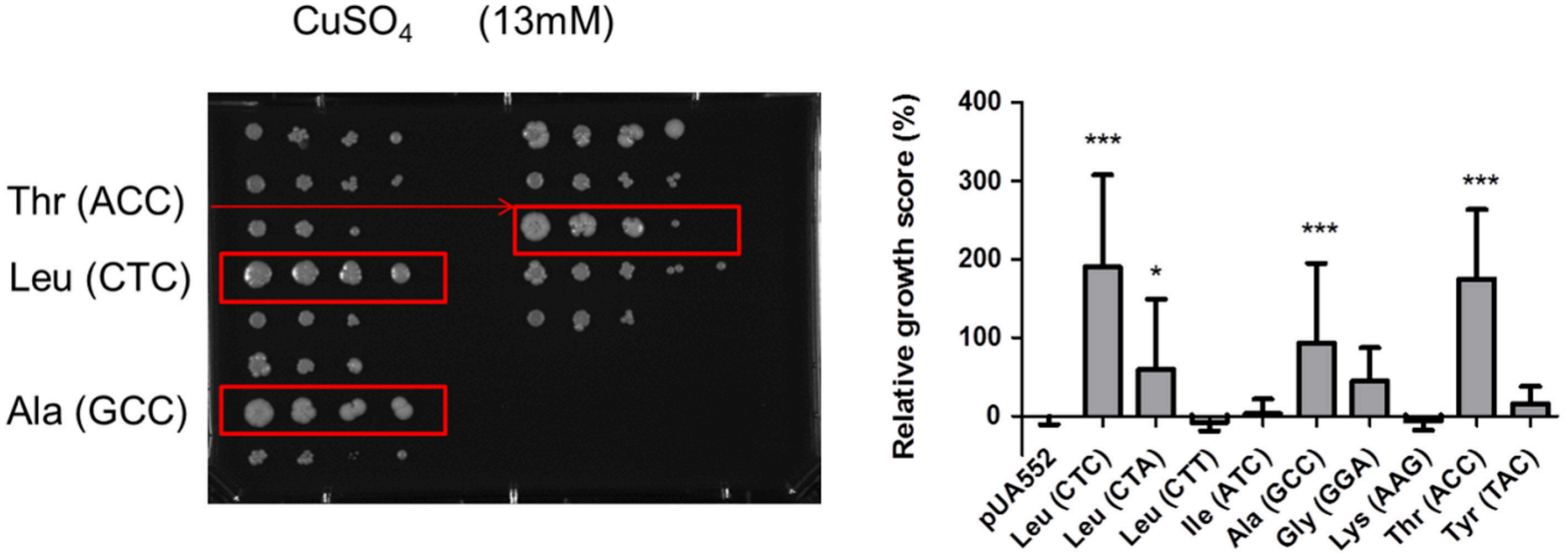
**Growth on YEPD supplemented with 13 mM of CuSO4**. Strains misincorporating serine in leucine CTC, alanine GCC and threonine ACC showed an advantageous growth phenotype. Data represent the mean ± standard deviation of triplicates of three independent clones (^***^p < 0.001; ^*^p < 0.05 one-way Anova post Dunnett's comparison test with CI of 95%, relative to the pUA552 control cells).

Previous studies also showed that yeast strains mistranslating the CUG codon have a competitive edge under specific stress conditions, namely high temperature, salts, heavy metals, and oxidants (Santos et al., 1999). To clarify whether mistranslation of different codons would also produce positive growth outcomes, a phenotypic screen assay testing 21 different conditions was carried out. These tests probed a broad spectrum of metabolic networks, including growth in different carbon sources, pH values (pH 5–8.6) and different temperatures (25–42°C) on agar plates supplemented with elevated concentrations of cations or antifungal agents (Supplementary Table 2). For drug and nutrient conditions the concentrations were calibrated to determine whether impairment or enhancement of growth relative to control could be observed. Independent isolates of each mistranslating strain were plated as a series of 10 fold dilutions on solid media, using a 96-pin bolt replicator (Caliper). After 3–5 days of incubation, plates were photographed using Axio Vision Software from Zeiss. All images were imported and processed using ImageJ software and scored for growth phenotypes by comparison to the control strains (pUA552) included in the test plates. This approach generated individual growth scores for each mutant on each growth medium (Figure 2). Hence, the scoring system measured the reduction or enhancement of growth relative to control (pUA552). The majority of the strains showed growth disadvantages at 37°C, except strains misreading the Leu CTA and Ala GCC codons. At 42°C the strains misreading the Lys AAG and Tyr TAC codons showed a slight growth advantage. Strains misreading Leu CTC and CTA and Thr ACC codons displayed advantageous growth in media containing CaCl_2_, CuSO_4_ (Figure 3), and galactose as single carbon source. Medium with pH 5.0 was deleterious to strains misreading Leu CTC and CTT, Ile ATC and Ala GCC codons. Although the changes in growth were idiosyncratic with each strain exhibiting a unique pattern of phenotypes, all strains had increased resistance to sorbitol (1.5 M) and guanidine hydro chloride (5 mM). When exposed to urea (25 mM), strains misreading Leu, Thr and Tyr codons also displayed advantageous growth. Finally, the sensitivity to oxidative stress was tested in media containing hydrogen peroxide (H_2_O_2_), and the strains misreading Leu CTC, Gly GGA, Lys AAG, and Thr ACC codons, grew faster than the control. Overall, there was variability between clones and stress conditions, nevertheless, strains expressing the misreading tRNAs performed better than the control strain in various growth conditions, supporting previous data from our laboratory on selective advantages of tRNA misreading under stress (Bezerra et al., 2013).

### Genome diversification

**Figure 4:**
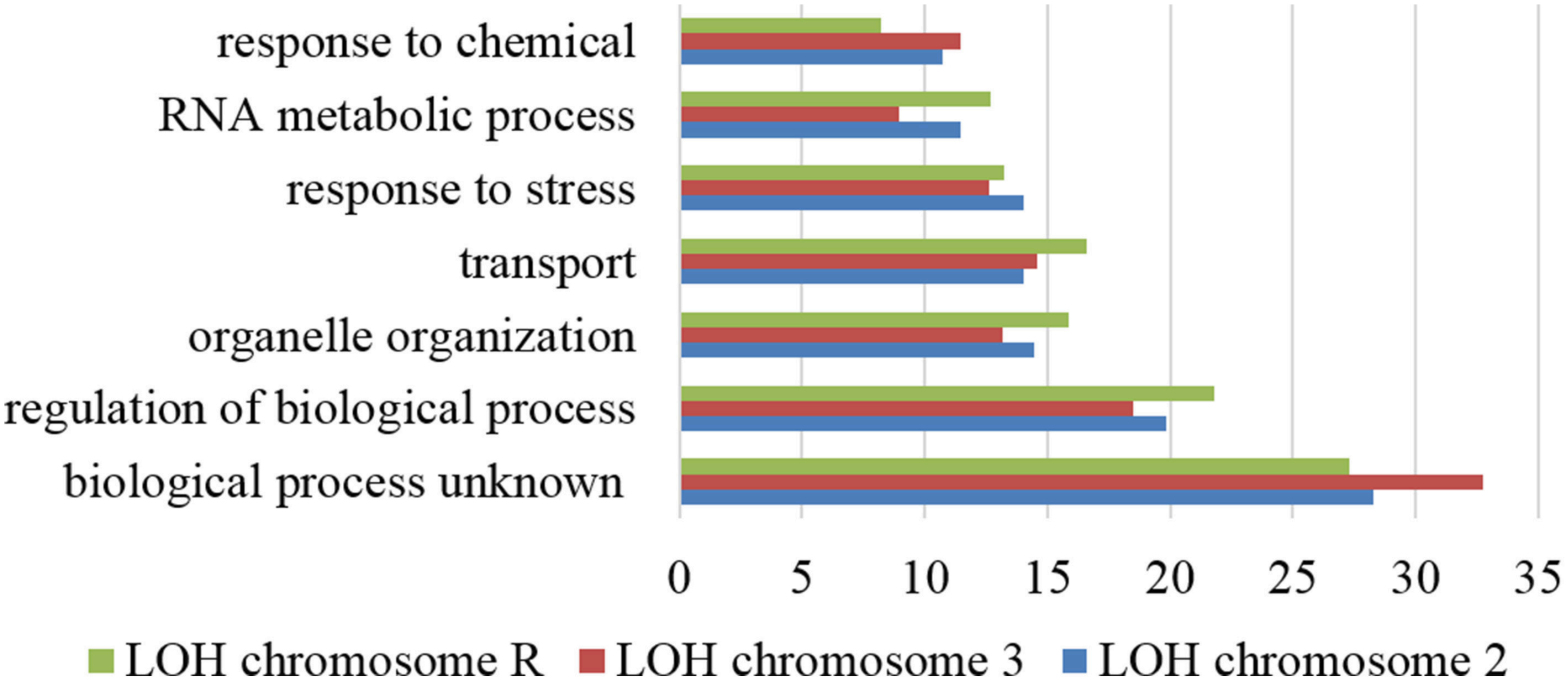
**Gene ontology process terms**. All genes affected by LOH regions of chromosome 2, 3, and R identified in the evolved strains. Data is relative to non-evolved strains. The graph represents the percentage of genes affected in each process above 10%.

To clarify if the proteome instability generated by the misreading tRNAs affected the genome, we have evolved the various strains for 100 generations and sequenced their genomes at the end of the evolution. Although read depth was relatively uniform across the genome in all strains, two evolved strains showed LOH relative to their unevolved progenitors. Indeed, strains misreading Leu CTC codons showed LOH in chromosome II (1,300,000–2,200,000) and chromosome III (100,000–900,000; Supplementary Figure 5), and the strain misreading the Ala-GCC codon showed LOH in chromosome R (300,000–1,500,000; Supplementary Figure 6). The affected region on chromosome II contains 428 ORFs, most of which are involved in unknown biological processes (28.3%), regulation of biological processes (19.9%), organelle organization (14.5%), transport (14.0%), and response to stress (14.0%; Figure 4). Noteworthy the cell adhesion process, which comprises 60 genes, was affected in 10 ORFs by this LOH. In chromosome III the affected region contains 357 ORFs. The majority of them are associated with unknown biological processes (32.8%), regulation of biological processes (18.5%), transport (14.6%), organelle organization (13.2%), and response to stress (12.6%). The affected region of chromosome R includes 560 ORFs and most of them are associated with unknown biological processes (27.3%), regulation of biological processes (21.8%), transport (16.6%), organelle organization (15.9%), and response to stress (13.2%).

**Figure 5:**
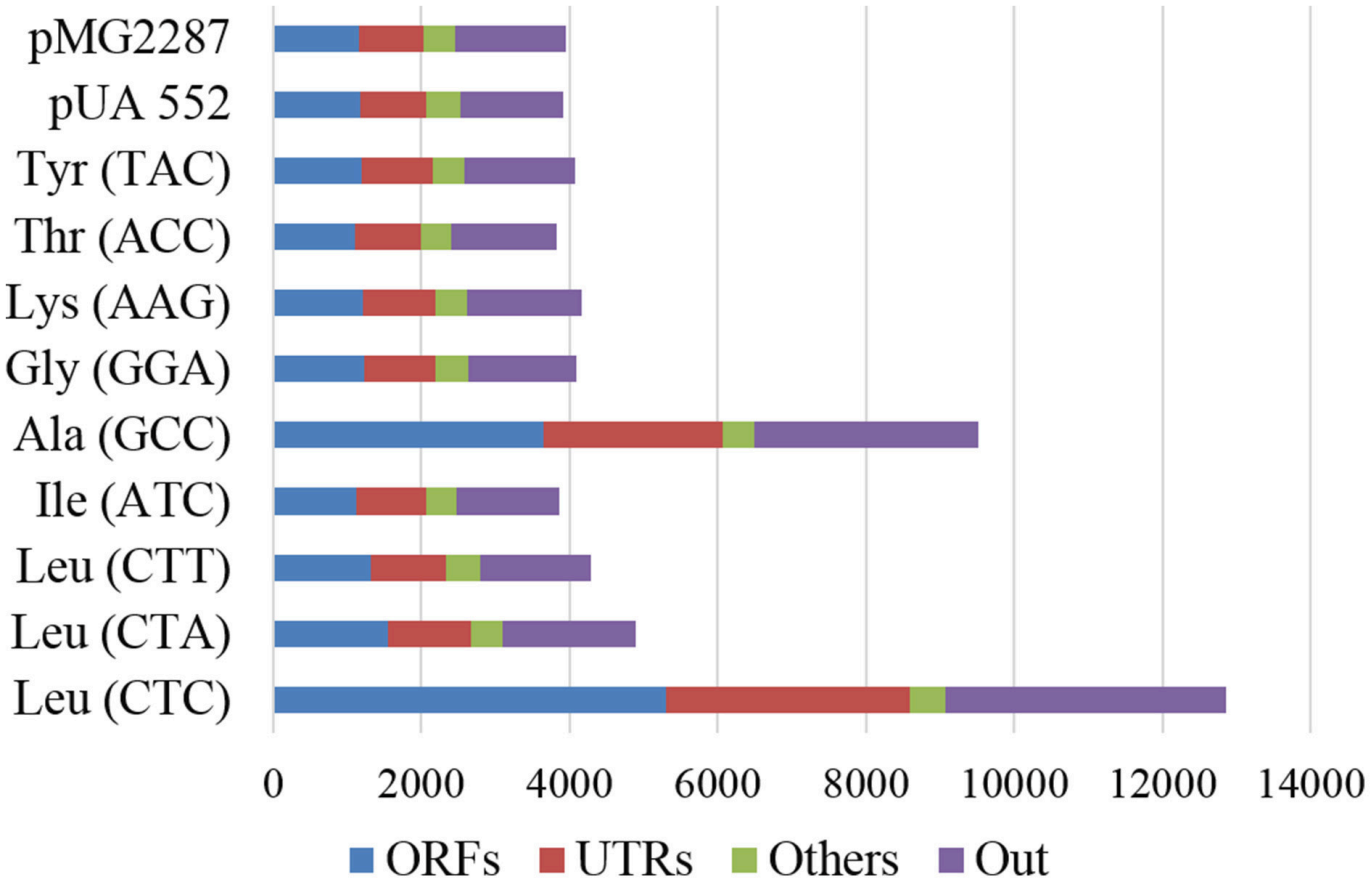
**Total number of SNP by genomic region**. ORFs (CDS, snoRNA, ncRNA, tRNA, and rRNA); Others (repeat-region, long-terminal repeat, retrotransposon, and centromeres); UTRs (blocked reading frame, snRNA, noncoding exon, and pseudogenes, 3′ UTR e 5′ UTR) and Out (regions not defined in ORFs, Others and UTRs). The data compares evolved and non-evolved strains.

Comparison of evolved with corresponding non-evolved parental strains, showed sharp increase of total number of SNPs during evolution. 3827 SNPs were detected in the strain misreading the ACC-Thr codon and 12855 in the strain misreading the Leu (CTC) (Figure 5). The number of strain specific SNPs (unique SNPs) followed similar trends varying between 268 and 7352 SNPs in strains misreading ACC-Thr and CTC-Thr codons, respectively (Supplementary Figure 7). The genomic regions that revealed higher occurrence of SNPs per Kbp of genome are classified as “Others” and include repeat-regions, long-terminal repeats, retrotransposon, and centromeres. The genome region defined as “UTRs” which includes blocked reading frames, snRNA, noncoding exon, and pseudogenes, 3′ UTR and 5′ UTR, contained one SNP per 1.5–5.1 kbp. The genome regions designated as “ORFs” showed the lowest SNP content (1.7–8.1 Kbp). It includes CDS, snoRNA, ncRNA, tRNA, and rRNA (Supplementary Table 4). Regarding nucleotide substitutions in protein coding genes (Supplementary Table 5), non-synonymous nucleotide substitutions (dN) ranged from 0.33 in CTC-Leu and Ala (GCC) to 0.37 in the ATC-Ile misreading strains. On the other hand, synonymous nucleotide substitutions ranged from 0.91 in the strain misreading GGA-Gly to 1.24 in strain misreading CTC-Leu. The substitution rates at non-synonymous and synonymous sites (dN/dS ratio) ranged from 0.27 in the strain misreading the CTC-Leu codon to 0.4 in strain misreading the GGA-Gly codon, suggesting that these strains are under purifying selection and replacement substitutions are been purged by natural selection.

**Figure 6:**
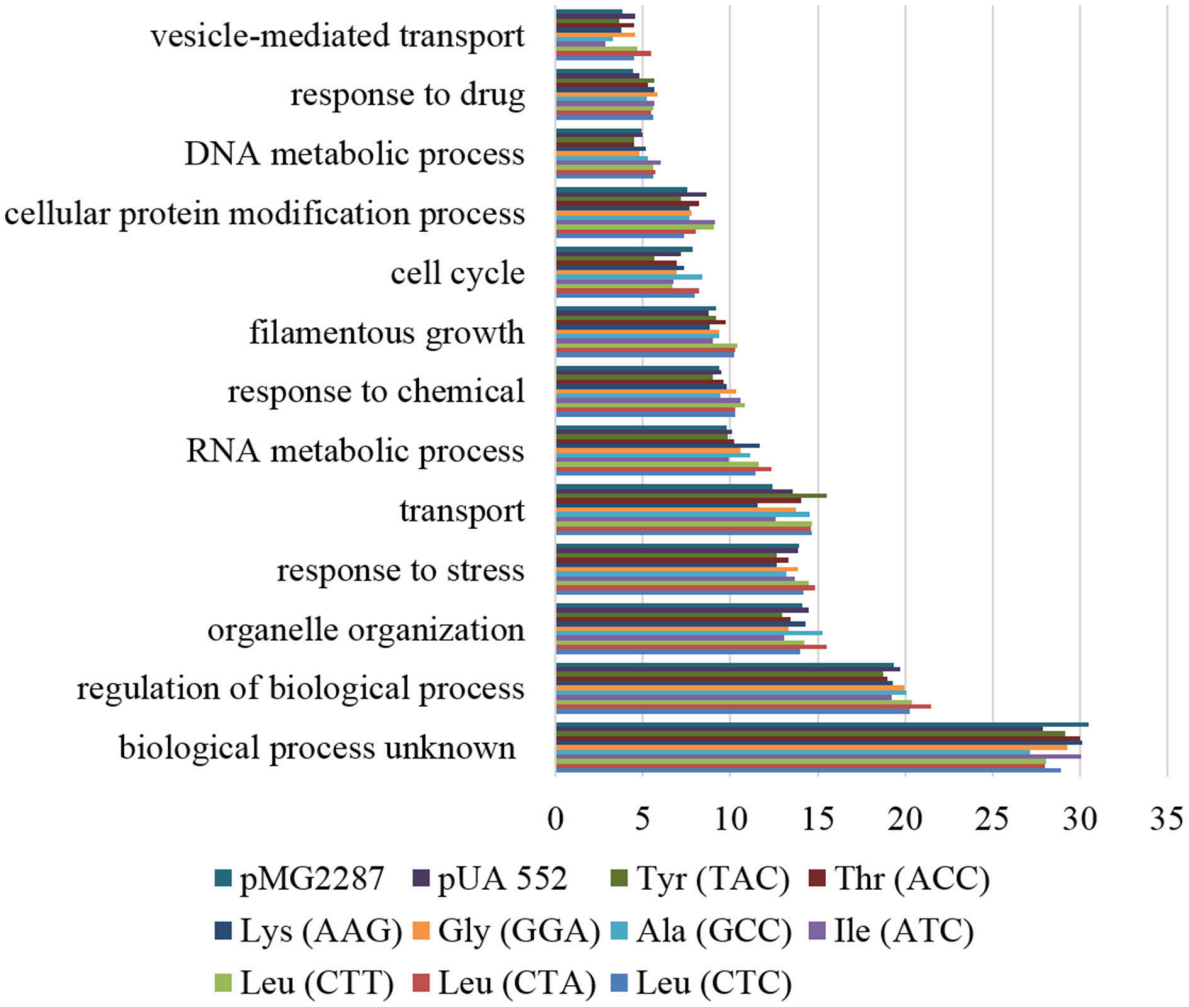
**Gene ontology process terms**. All genes affected by SNPs identified in the evolved strains, relative to non-evolved strains. The graph represents the percentage of genes affected in each process above 5%.

Enrichment analysis of ORFs containing SNPs, highlighted similar functions affected in all strains. The processes with higher number of affected ORFs belong to unknown biological processes (27.14–30.52%), followed by regulation of biological processes (18.73–21.49%), organelle organization, response to stress (13.07–15.54%), transport (11.53–15.49%), and RNA metabolic processes (9.82–12.34%; Figure 6). Scoring and comparison of the ORFs containing SNPs showed that orf19.2930 and orf19.1834 were shared by all but the control strains. Orf19.2930 is a predicted translation initiation factor and is repressed under spider biofilm medium, however orf19.1834 is uncharacterized. A SNP present at position 1625 of the deneddylase gene *JAB1* (orf19.3371) was shared by all but the control strain. This gene codes for a subunit of the complex COP9 signalosome and promotes the deneddylation of cullin (Cdc53p) (Sela et al., 2012), suggesting that deneddylation is relevant for adaptation to mistranslation.

## Discussion

To test if the known tolerance of *C. albicans* to CUG ambiguity could be extended to other codons, we have constructed a series of chimeric Serine tRNAs that are able to decode various non-cognate codons. These tRNAs are similar to the WT Ser tRNA that misincorporates Ser at Leu-CUG sites on a proteome wide scale (${\text{tRNA}}_{\text{CAG}}\text{Ser}$). This was possible because, as demonstrated by the ${\text{tRNA}}_{\text{CAG}}\text{Ser}$ and other *in vitro* data, the eukaryotic seryl-tRNA synthetase (SerRS) does not recognize the anticodon of Ser tRNAs (Cusack et al., 1996; Lenhard et al., 1999). Indeed, Geslain and colleagues demonstrated that chimeric Ser tRNAs are readily aminoacylated with Ser by the SerRS and misincorporate this amino acid at the non-cognate codons specified by their anticodons, mutagenizing the entire proteome (Geslain et al., 2010). Expression of these tRNAs creates a new situation in the cell where targeted codons are decoded by both chimeric Ser tRNAs and WT tRNAs, producing ambiguous codons. This has important consequences for the cell. Indeed, Bloom-Ackermann and coworkers altered the tRNA pool of *S. cerevisiae* by engeneering a tRNA deletion library and showed that erasing single copy tRNA genes from the genome is sufficient to trigger proteotoxic stress responses, linking aberrant tRNA decoding and protein misfolding. Conversely, deleting representative tRNAs from multi-copy tRNA gene families resulted in milder responses due to compensatory effects of tRNA up-regulation (Bloom-Ackermann et al., 2014).

Amino acid misincorporations normally occur at frequencies of 10^−3^–10^−4^/translated codon (Loftfield and Vanderjagt, 1972), but *C. albicans* is very different. Our previous studies showed that 98% of Leu or 99% of Ser can be incorporated at CUG sites without killing the cell, i.e., this codon has two true identities. We have also demonstrated that *C. albicans* proteins are not affected by this unique phenomenon due to an exquisite evolutionary fine tuning of the localization of CUG encoded residues on protein structure (Rocha et al., 2011). However, nothing is known about the tolerance of other codons to ambiguity. The present study addressed this question. The data show that *C. albicans* can indeed tolerate Ser misincorporation at other codons. We have not quantified the levels of such misincorporation and we cannot exclude compensatory effects associated with upregulation of other tRNAs, in any case, the data provides a strong indication that *C. albicans* is far more tolerant to codon ambiguity that yeast, an organism whose tolerance to codon ambiguity has also been studied in detail (Silva et al., 2007).

In any case, the expression of our mutant misreading tRNAs decreased *C. albicans* transformation efficiency and growth rate (Supplementary Figures 2, 3). This toxicity is likely related with widespread protein mutagenesis caused by Ser misincorporation at multiple codon sites. The differential phenotypic effects observed may be associated with chemical differences between the amino acids substitutions studied. However, codon usage and localization of misincorporated Ser may also explain those differences. Indeed, Trp, Met, Phe, Ile, Val, Leu, Ala, and Tyr are frequently buried in proteins cores while Thr, Ser, Asn, Glu, Arg, Asp, Glu, and Lys are often exposed on proteins surfaces. Gly and His could be buried or present on protein surfaces (Fiser et al., 1996). The misreading tRNAs that had stronger effects on cell transformation targeted Leu, Ile, and Thr codons, which is in agreement with the results of Geslain and colleagues showing that Ser misincorporation at Ile codons has a strong inhibitory effect on cell division (Geslain et al., 2010). The transformation efficiency of the plasmid carrying the WT tRNA gene (pUA552) was also lower than the plasmid alone (pMG2287), suggesting that increasing copy number of WT tRNA genes is toxic. However, growth rate and ^35^S-Met incorporation of the strain transformed with the WT tRNA was higher than the strain transformed with the empty plasmid (pMG2287), suggesting that tRNA overexpression may increase translational activity (Zhou et al., 2009). Misreading of Leu codons suggested that codon usage is an important modulator of amino acid misincorporation toxicity. Indeed, toxicity was weaker in strains expressing misreading tRNAs that decoded frequently used codons and stronger in more rarely used codons; in the following order CTC>CTA>>CTT (codon usage of 2.6, 4.4, and 10.2 per1000 codons, respectively). We propose that the ratio of recombinant vs. WT tRNA is responsible for these effects.

Temperature (37 and 42°C) had a strong negative effect on growth rate in almost all recombinant strains, which may be explained by synergistic effects of proteome disruption, accumulation of misfolded proteins and saturation of Hsp 70, Hsp 90, and Hsp 100 chaperones (Burnie et al., 2006). The latter are particularly relevant due to protein protection against thermal stress, folding repair, and targetting unrecoverable proteins for degradation (Panaretou and Zhai, 2008). We hypothesize that the erroneous proteins synthesized by tRNA misreading sequester Hsps and prevent their rapid recycling; a phenotype that is exacerbated at higher temperature. Interestingly, all recombinant strains had higher fitness in 1.5 M sorbitol, 5 mM guanidine hydrochloride and 25 mM urea. Since guanidine hydrochloride and urea are protein denaturation agents and have a strong negative effect on growth rate we hypothesize that the stress response is strongly induced in the mistranslating strains, pre-adapting them to tolerance to protein misfolding agents. These data is fully concordant with previous studies from our laboratory showing that codon ambiguity produces phenotypic diversity and allows cells to proliferate in ecological niches where WT cells are unable to grow or grow poorly (Pezo et al., 2004; Ruan et al., 2008; Bezerra et al., 2013).

Experimental evolution followed by genome sequencing showed that proteome instability caused by tRNA misreading has a strong impact on the *C. albicans* genome, supporting previous results from our laboratory (Bezerra et al., 2013). Indeed, the LOH events observed in the strains misreading CTC-Leu and GCC-Ala codons are in agreement with our previous studies. The strain misreading CTC-Leu codons had higher number of SNPs, suggesting that SNP accumulation is directly proportional to the level of toxicity induced by mistranslation. These genomic alterations confirm that mRNA mistranslation is a strong modulator of genome evolution and that misreading tRNAs are sufficient to speed up genome evolution under conditions where cell division rate is low. The SNP identified in the *JAB1* gene is most interesting. Its accumulation in all recombinant strains indicates that neddylation is relevant for adaptation to mistranslation and protein misfolding/aggregation. Since the strain with higher level of protein aggregation, namely the CTC-Leu misreading strain also had 5 SNPs at positions 1124, 1265, 1526, 1604, and 2060 in the *CDC53* ORF, which encodes a cullin scaffold subunit of the SCF ubiquitin-ligase complex, whose depletion increases filamentous growth and premature cell death (Trunk et al., 2009), it is likely that filamentation of the tRNA misreading strains is somehow related with inactivation of CDC53 or deregulation of the ubiquitin-proteasome pathway. Alternatively, neddylation may play a role in adaptation to mistranslation through DNA repair (Chung and Dellaire, 2015).

In conclusion, our study shows that *C. albicans* tolerance to codon ambiguity is widespread. This fungus adapts rather rapidly to the loss of fitness induced by misreading tRNAs through accumulation of compensatory genomic mutations. Comparison of the mistranslation data produced by our laboratory over the years in both yeast and *C. albicans*, shows that the former adapts to mistranslation through large chromosomal rearrangements, increased protein degradation and synthesis, while the latter accumulates SNP in a wide variety of genes, including genes involved in post-translational neddylation of proteins.

## Author contributions

MS designed the study. JS and AB performed experiments. IG and MB sequenced strains. HA, GM, IG and MB analyzed sequencing data. JS, AB, and MS interpreted data. JS drafted the paper. JS and MS wrote the manuscript.

### Conflict of interest statement

The authors declare that the research was conducted in the absence of any commercial or financial relationships that could be construed as a potential conflict of interest.
