# Supplementary material for: The Fungus Candida albicans Tolerates Ambiguity at Multiple Codons
Source: Front Microbiol. 2016 Mar 31;7:401. doi: 10.3389/fmicb.2016.00401 (PMC4814463; doi:10.3389/fmicb.2016.00401)
Supplement: Supplementary file 1 [file Table1.DOCX]

**Supplementary Table 1: List of plasmids and primer sequences used to mutate the tRNA^Ser^ anticodon.** The new anticodons obtained by mutagenesis and corresponding codons and amino acids are also indicated

| **Plasmid Name** | **Primer Name** | **Primer Sequence (5'->3')** | **Anticodon (5'->3')** | **Codon (5'->3')** | **Amino acid** |
| --- | --- | --- | --- | --- | --- |
| pUA533 | oUA1719 | TAAGGCGACAGACGTAGAATCTGTTGGGCTC | UAG | CTA | Leucine |
|  | oUA1720 | GAGCCCAACAGATTCTACGTCTGTCGCCTTA |  |  |  |
| pUA534 | oUA1721 | TAAGGCGACAGACGGAGAATCTGTTGGGCTC | GAG | CTC | Leucine |
|  | oUA1722 | GAGCCCAACAGATTCTCCGTCTGTCGCCTTA |  |  |  |
| pUA535 | oUA1723 | TAAGGCGACAGACGAAGAATCTGTTGGGCTC | AAG | CTT | Leucine |
|  | oUA1724 | GAGCCCAACAGATTCTTCGTCTGTCGCCTTA |  |  |  |
| pUA536 | oUA1725 | TAAGGCGACAGACGGATAATCTGTTGGGCTC | GAU | ATC | Isoleucine |
|  | oUA1726 | GAGCCCAACAGATTATCCGTCTGTCGCCTTA |  |  |  |
| pUA537 | oUA1727 | TAAGGCGACAGACGGGCAATCTGTTGGGCTC | GGC | GCC | Alanine |
|  | oUA1728 | GAGCCCAACAGATTGCCCGTCTGTCGCCTTA |  |  |  |
| pUA540 | oUA1739 | TAAGGCGACAGACGTCCAATCTGTTGGGCTC | UCC | GGA | Glycine |
|  | oUA1740 | GAGCCCAACAGATTGGACGTCTGTCGCCTTA |  |  |  |
| pUA542 | oUA1747 | TAAGGCGACAGACGCTTAATCTGTTGGGCTC | CUU | AAG | Lysine |
|  | oUA1748 | GAGCCCAACAGATTAAGCGTCTGTCGCCTTA |  |  |  |
| pUA544 | oUA1751 | TAAGGCGACAGACGGGTAATCTGTTGGGCTC | GGU | ACC | Threonine |
|  | oUA1752 | GAGCCCAACAGATTACCCGTCTGTCGCCTTA |  |  |  |
| pUA546 | oUA1755 | TAAGGCGACAGACGGTAAATCTGTTGGGCTC | GUA | TAC | Tyrosine |
|  | oUA1756 | GAGCCCAACAGATTTACCGTCTGTCGCCTTA |  |  |  |
