# Supplementary material for: The Fungus Candida albicans Tolerates Ambiguity at Multiple Codons
Source: Front Microbiol. 2016 Mar 31;7:401. doi: 10.3389/fmicb.2016.00401 (PMC4814463; doi:10.3389/fmicb.2016.00401)
Supplement: Supplementary file 2 [file Table2.DOCX]

**Supplementary Table 2: List of stressor compound with respective concentration and conditions used.**

| **Assay** | **Stress compound** | **Concentration** | **Base medium** | **Growth temperature** |
| --- | --- | --- | --- | --- |
| Control |  |  | YEPD/ MM-Uri | 30ºC |
| Temperature |  |  | MM-Uri | 25ºC |
|  |  |  |  | 37ºC |
|  |  |  |  | 42ºC |
| Elevated cation concentration | Calcium chloride | 300 mM | MM-Uri | 30ºC |
|  | Sodium chloride | 1.3 M |  |  |
| Carbon source | Carbon source absent |  | MM-Uri without glucose | 30ºC |
|  | Galactose | 2% (w/v) |  |  |
|  | Glycerol | 3% (w/v) |  |  |
|  | Ethanol | 2% (w/v) |  |  |
| Protein denaturation | Guanidine HCl | 5 mM | MM-Uri | 30ºC |
|  | Urea | 25 mM |  |  |
| pH value | pH 5.0 | pH 5.0 | YEPD + buffered glycine | 30ºC |
|  | pH 8.6 | pH 8.6 |  |  |
| Stress | Calcofluor White | 20 µM | MM-Uri | 30ºC |
|  | Sorbitol | 1.5 M | MM-Uri |  |
|  | Caffeíne | 15 mM | MM-Uri |  |
|  | EDTA | 0.75 mM | YEPD/ MM-Uri |  |
|  | Hydrogen Peroxyde | 6.0 mM | YEPD/ MM-Uri |  |
|  | CuSO_4_ | 13 mM | YEPD |  |
| Antifungal resitance | Fluconazole | 0.5 µg/ml | MM-Uri | 30ºC |
