# Supplementary material for: The Fungus Candida albicans Tolerates Ambiguity at Multiple Codons
Source: Front Microbiol. 2016 Mar 31;7:401. doi: 10.3389/fmicb.2016.00401 (PMC4814463; doi:10.3389/fmicb.2016.00401)
Supplement: Supplementary file 3 [file Table3.docx]

**Supplementary Table 3: Amino acids characteristics and codon usage.** Table adapted from Haig and Hurst 1991 (Haig and Hurst, 1991).

| **Amino acid** | **Codon (5'->3')** | **codon usage (frequency per 1000)** | **Hydropathy** | **Molecular volume (Å^3^)** | **Molecular Weight (D)** |
| --- | --- | --- | --- | --- | --- |
| Alanine (Ala) | GCC | 11.7 | 1.8 | 88.6 | 89 |
| Glycine (Gly) | GGA | 13.7 | -0.4 | 60.1 | 75 |
| Isoleucine (Ile) | ATC | 13.5 | 4.5 | 166.7 | 131 |
| Leucine (Leu) | CTC | 2.6 | 3.8 | 166.7 | 131 |
|  | CTA | 4.4 |  |  |  |
|  | CTT | 10.2 |  |  |  |
| Lysine (Lys) | AAG | 18.3 | -3.9 | 168.6 | 146 |
| Serine (Ser) | TCA | 26.4 | -0.8 | 89 | 105 |
| Threonine (Thr) | ACC | 13.5 | -0.7 | 116.1 | 119 |
| Tyrosine (Tyr) | TAC | 10.4 | -1.3 | 193.6 | 181 |

**Reference List**

Haig, D., and Hurst, L. D. (1991). A quantitative measure of error minimization in the genetic code. *J. Mol. Evol.* 33, 412–7.
