# Supplementary material for: The Fungus Candida albicans Tolerates Ambiguity at Multiple Codons
Source: Front Microbiol. 2016 Mar 31;7:401. doi: 10.3389/fmicb.2016.00401 (PMC4814463; doi:10.3389/fmicb.2016.00401)
Supplement: Supplementary file 4 [file Table4.DOCX]

**Supplementary Table 4: Total number of SNP by genomic region.** SNP detected by Kbp**.** ORFs (CDS, snoRNA, ncRNA, tRNA and rRNA); Others (i repeat-region, long-terminal repeat, retrotransposon and centromeres); UTRs (blocked reading frame, snRNA, noncoding exon and pseudogenes, 3' UTR e 5' UTR) and Out (regions not defined in ORFs, Others and UTRs). Comparison between strains not evolved and respective evolved strains

| **Strain** | **ORFs** | **UTRs** | **Others** | **Out** |
| --- | --- | --- | --- | --- |
| Leu (CTC) | 1.7 | 1.5 | 0.5 | 0.9 |
| Leu (CTA) | 5.9 | 4.2 | 0.6 | 1.9 |
| Leu (CTT) | 6.9 | 4.6 | 0.5 | 2.3 |
| Ile (ATC) | 8.1 | 5.1 | 0.6 | 2.4 |
| Ala (GCC) | 2.5 | 2.0 | 0.5 | 1.1 |
| Gly (GGA) | 7.4 | 5.0 | 0.5 | 2.3 |
| Lys (AAG) | 7.5 | 4.9 | 0.5 | 2.2 |
| Thr (ACC) | 8.2 | 5.3 | 0.6 | 2.4 |
| Tyr (TAC) | 7.6 | 5.0 | 0.5 | 2.3 |
| pUA 552 | 7.7 | 5.3 | 0.5 | 2.4 |
| pMG2287 | 7.8 | 5.5 | 0.6 | 2.3 |
