# Supplementary material for: The Fungus Candida albicans Tolerates Ambiguity at Multiple Codons
Source: Front Microbiol. 2016 Mar 31;7:401. doi: 10.3389/fmicb.2016.00401 (PMC4814463; doi:10.3389/fmicb.2016.00401)
Supplement: Supplementary file 5 [file Table5.DOCX]

**Supplementary Table 5: Values of DN, DS an DN/DS ratio for genome of *C. albicans* strains evolved for 100 generations and compared with initial strain.** DN (synonymous substitutions per substitution site), DS (non-synonymous substitution per non-substitution site).

| **Strain** | **DN** | **DS** | **DN/DS** |
| --- | --- | --- | --- |
| Leu (CTC) | 0.33 | 1.24 | 0.27 |
| Leu (CTA) | 0.35 | 1.02 | 0.34 |
| Leu (CTT) | 0.35 | 1.01 | 0.35 |
| Ile (ATC) | 0.37 | 0.94 | 0.39 |
| Ala (GCC) | 0.33 | 1.14 | 0.29 |
| Gly (GGA) | 0.36 | 0.91 | 0.40 |
| Lys (AAG) | 0.36 | 1.01 | 0.35 |
| Thr (ACC) | 0.35 | 1.14 | 0.30 |
| Tyr (TAC) | 0.36 | 0.93 | 0.38 |
| pUA 552 | 0.35 | 1.01 | 0.35 |
| pMG2287 | 0.36 | 0.94 | 0.38 |
