# Supplementary material for: The Fungus Candida albicans Tolerates Ambiguity at Multiple Codons
Source: Front Microbiol. 2016 Mar 31;7:401. doi: 10.3389/fmicb.2016.00401 (PMC4814463; doi:10.3389/fmicb.2016.00401)
Supplement: Supplementary file 6 [file DataSheet1.DOCX]

Supplementary Material

The fungus *Candida albicans* tolerates high level of ambiguity at multiple codons (normal version)

João Salvador Simões^1^, Ana Rita Bezerra^1^, Gabriela Moura^1^, Hugo Araújo^1^, Ivo Gut^2^, Mónica Bayes^2^ and Manuel A. S. Santos^1^

*** Correspondence:** Manuel Santos, [msantos@ua.pt](mailto:msantos@ua.pt)

## Supplementary Figures


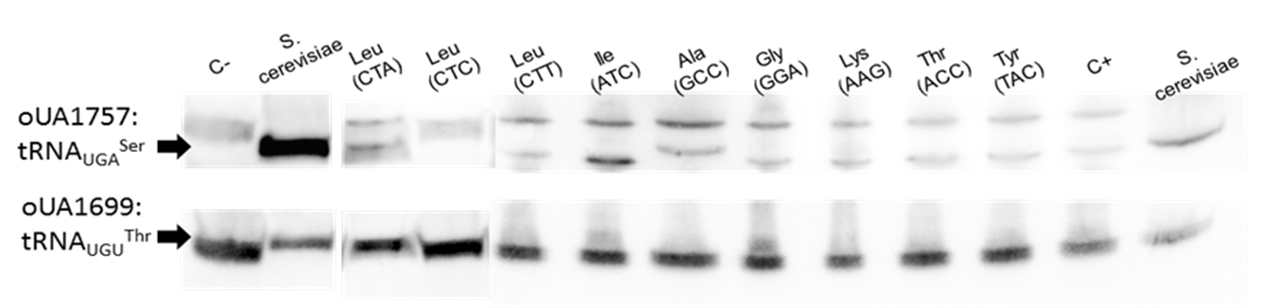


**Supplementary Figure 1: Northern blot analysis of the mutant tRNAs.** 50 µg of total RNA were resolved at room temperature on 15% polyacrylamide (40% Acril:Bis) gels containing 8 M urea. Probe oUA1757 labeled with ɣ-^32^P-ATP was used to detect tRNA_UGA_^Ser^, probe oUA1699 labeled with ɣ-^32^P-ATP was used to detect the internal control tRNA_UGU_^Thr^.

**Supplementary Figure 2: Transformation efficiency.** Transformation efficiency was determined using at least 3 independent transformations. Values obtained were normalized relative to the control PM2287 strain (%). Data are mean + s.d., and shows sharp reduction in the transformation efficiency of all misreading tRNA genes, relative to the control.

**Supplementary Figure 3: Growth rate of mistranslating *C. albicans* strains**. The data shows decreased growth rate in all strains, however strains misincorporating serine at chemically distinct sites show sharper negative effects. Data represents growth rate mean + s.d. of triplicates of 3 different clones, normalized to the pUA552 control. Data statistical analysis one-way ANOVA was performed followed by a Dunnet test with CI 95% relative to pUA552 (***p<0.001, **p<0.01, *p<0.05).

**Supplementary Figure 4: Effect of mistranslation on protein synthesis rate**. *C. albicans* cells were collected in exponential growth phase and incubated for 20 min in media lacking methionine. Cells were labelled with [^35^S]-Met for 8 min and protein synthesis was then stopped by adding cycloheximide. Data represents mean incorporation of [^35^S]-Met on new synthesized protein + s.d. of triplicates of 3 different clones, normalized with pUA552. Statistical analysis was carried out using one-way ANOVA followed by a Dunnet test with CI 95% relative to pUA552 (***p<0.001, **p<0.01, *p<0.05).


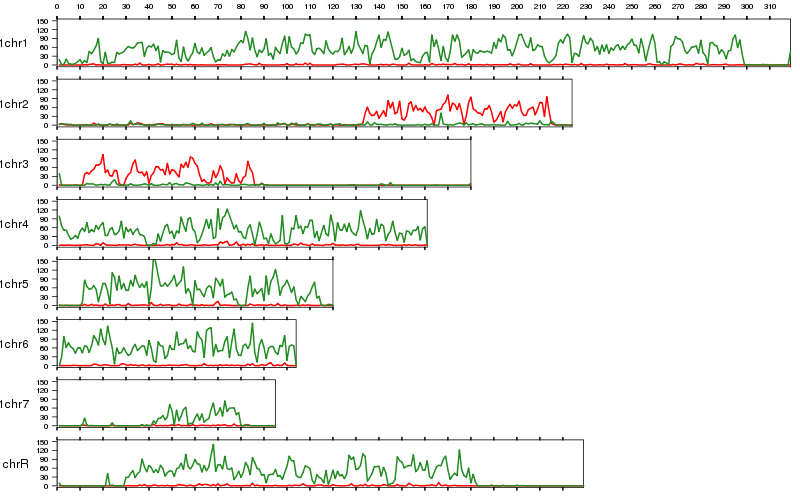


**Supplementary Figure 5:** Genomic analysis of the leu CTC (not evolved strain) and evolved strains without Amphotericin shows LOH in a region of chromosome 2 and 3 in leu(CTC). SNPs per kilobase are shaded green; density of LOHSNPs is in red, and gray vertical lines indicate the major repeat sequence.


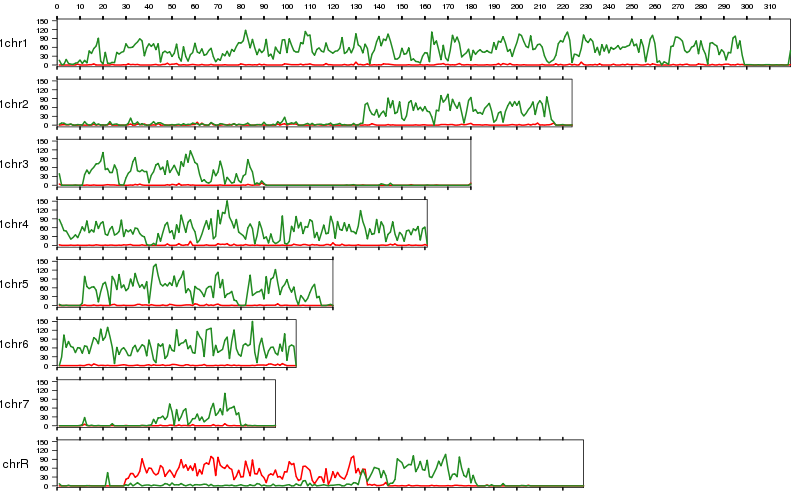


**Supplementary Figure 6:** Genomic analysis of the control (not evolved strain) and misincorporating strains shows LOH in a region of chromosome R in ala (GCC) . SNPs per kilobase are shaded green; density of LOHSNPs is in red, and gray vertical lines indicate the major repeat sequence.

**Supplementary Figure 7: Total number of unique SNP.** Comparison between strains not evolved and respective evolved strains

## Supplementary Tables

**Supplementary Table 1: List of plasmids and primer sequences used to mutate the tRNA^Ser^ anticodon.** The new anticodons obtained by mutagenesis and corresponding codons and amino acids are also indicated

| **Plasmid Name** | **Primer Name** | **Primer Sequence (5'->3')** | **Anticodon (5'->3')** | **Codon (5'->3')** | **Amino acid** |
| --- | --- | --- | --- | --- | --- |
| pUA533 | oUA1719 | TAAGGCGACAGACGTAGAATCTGTTGGGCTC | UAG | CTA | Leucine |
|  | oUA1720 | GAGCCCAACAGATTCTACGTCTGTCGCCTTA |  |  |  |
| pUA534 | oUA1721 | TAAGGCGACAGACGGAGAATCTGTTGGGCTC | GAG | CTC | Leucine |
|  | oUA1722 | GAGCCCAACAGATTCTCCGTCTGTCGCCTTA |  |  |  |
| pUA535 | oUA1723 | TAAGGCGACAGACGAAGAATCTGTTGGGCTC | AAG | CTT | Leucine |
|  | oUA1724 | GAGCCCAACAGATTCTTCGTCTGTCGCCTTA |  |  |  |
| pUA536 | oUA1725 | TAAGGCGACAGACGGATAATCTGTTGGGCTC | GAU | ATC | Isoleucine |
|  | oUA1726 | GAGCCCAACAGATTATCCGTCTGTCGCCTTA |  |  |  |
| pUA537 | oUA1727 | TAAGGCGACAGACGGGCAATCTGTTGGGCTC | GGC | GCC | Alanine |
|  | oUA1728 | GAGCCCAACAGATTGCCCGTCTGTCGCCTTA |  |  |  |
| pUA540 | oUA1739 | TAAGGCGACAGACGTCCAATCTGTTGGGCTC | UCC | GGA | Glycine |
|  | oUA1740 | GAGCCCAACAGATTGGACGTCTGTCGCCTTA |  |  |  |
| pUA542 | oUA1747 | TAAGGCGACAGACGCTTAATCTGTTGGGCTC | CUU | AAG | Lysine |
|  | oUA1748 | GAGCCCAACAGATTAAGCGTCTGTCGCCTTA |  |  |  |
| pUA544 | oUA1751 | TAAGGCGACAGACGGGTAATCTGTTGGGCTC | GGU | ACC | Threonine |
|  | oUA1752 | GAGCCCAACAGATTACCCGTCTGTCGCCTTA |  |  |  |
| pUA546 | oUA1755 | TAAGGCGACAGACGGTAAATCTGTTGGGCTC | GUA | TAC | Tyrosine |
|  | oUA1756 | GAGCCCAACAGATTTACCGTCTGTCGCCTTA |  |  |  |

**Supplementary Table 2: List of stressor compound with respective concentration and conditions used.**

| **Assay** | **Stress compound** | **Concentration** | **Base medium** | **Growth temperature** |
| --- | --- | --- | --- | --- |
| Control |  |  | YEPD/ MM-Uri | 30ºC |
| Temperature |  |  | MM-Uri | 25ºC |
|  |  |  |  | 37ºC |
|  |  |  |  | 42ºC |
| Elevated cation concentration | Calcium chloride | 300 mM | MM-Uri | 30ºC |
|  | Sodium chloride | 1.3 M |  |  |
| Carbon source | Carbon source absent |  | MM-Uri without glucose | 30ºC |
|  | Galactose | 2% (w/v) |  |  |
|  | Glycerol | 3% (w/v) |  |  |
|  | Ethanol | 2% (w/v) |  |  |
| Protein denaturation | Guanidine HCl | 5 mM | MM-Uri | 30ºC |
|  | Urea | 25 mM |  |  |
| pH value | pH 5.0 | pH 5.0 | YEPD + buffered glycine | 30ºC |
|  | pH 8.6 | pH 8.6 |  |  |
| Stress | Calcofluor White | 20 µM | MM-Uri | 30ºC |
|  | Sorbitol | 1.5 M | MM-Uri |  |
|  | Caffeíne | 15 mM | MM-Uri |  |
|  | EDTA | 0.75 mM | YEPD/ MM-Uri |  |
|  | Hydrogen Peroxyde | 6.0 mM | YEPD/ MM-Uri |  |
|  | CuSO_4_ | 13 mM | YEPD |  |
| Antifungal resitance | Fluconazole | 0.5 µg/ml | MM-Uri | 30ºC |

**Supplementary Table 3: Amino acids characteristics and codon usage.** Table adapted from Haig and Hurst 1991 (32).

| **Amino acid** | **Codon (5'->3')** | **codon usage (frequency per 1000)** | **Hydropathy** | **Molecular volume (Å^3^)** | **Molecular Weight (D)** |
| --- | --- | --- | --- | --- | --- |
| Alanine (Ala) | GCC | 11.7 | 1.8 | 88.6 | 89 |
| Glycine (Gly) | GGA | 13.7 | -0.4 | 60.1 | 75 |
| Isoleucine (Ile) | ATC | 13.5 | 4.5 | 166.7 | 131 |
| Leucine (Leu) | CTC | 2.6 | 3.8 | 166.7 | 131 |
|  | CTA | 4.4 |  |  |  |
|  | CTT | 10.2 |  |  |  |
| Lysine (Lys) | AAG | 18.3 | -3.9 | 168.6 | 146 |
| Serine (Ser) | TCA | 26.4 | -0.8 | 89 | 105 |
| Threonine (Thr) | ACC | 13.5 | -0.7 | 116.1 | 119 |
| Tyrosine (Tyr) | TAC | 10.4 | -1.3 | 193.6 | 181 |

**Supplementary Table 4: Total number of SNP by genomic region.** SNP detected by Kbp**.** ORFs (CDS, snoRNA, ncRNA, tRNA and rRNA); Others (i repeat-region, long-terminal repeat, retrotransposon and centromeres); UTRs (blocked reading frame, snRNA, noncoding exon and pseudogenes, 3' UTR e 5' UTR) and Out (regions not defined in ORFs, Others and UTRs). Comparison between strains not evolved and respective evolved strains

| **Strain** | **ORFs** | **UTRs** | **Others** | **Out** |
| --- | --- | --- | --- | --- |
| Leu (CTC) | 1.7 | 1.5 | 0.5 | 0.9 |
| Leu (CTA) | 5.9 | 4.2 | 0.6 | 1.9 |
| Leu (CTT) | 6.9 | 4.6 | 0.5 | 2.3 |
| Ile (ATC) | 8.1 | 5.1 | 0.6 | 2.4 |
| Ala (GCC) | 2.5 | 2.0 | 0.5 | 1.1 |
| Gly (GGA) | 7.4 | 5.0 | 0.5 | 2.3 |
| Lys (AAG) | 7.5 | 4.9 | 0.5 | 2.2 |
| Thr (ACC) | 8.2 | 5.3 | 0.6 | 2.4 |
| Tyr (TAC) | 7.6 | 5.0 | 0.5 | 2.3 |
| pUA 552 | 7.7 | 5.3 | 0.5 | 2.4 |
| pMG2287 | 7.8 | 5.5 | 0.6 | 2.3 |

**Supplementary Table 5: Values of DN, DS an DN/DS ratio for genome of *C. albicans* strains evolved for 100 generations and compared with initial strain.** DN (synonymous substitutions per substitution site), DS (non-synonymous substitution per non-substitution site).

| **Strain** | **DN** | **DS** | **DN/DS** |
| --- | --- | --- | --- |
| Leu (CTC) | 0.33 | 1.24 | 0.27 |
| Leu (CTA) | 0.35 | 1.02 | 0.34 |
| Leu (CTT) | 0.35 | 1.01 | 0.35 |
| Ile (ATC) | 0.37 | 0.94 | 0.39 |
| Ala (GCC) | 0.33 | 1.14 | 0.29 |
| Gly (GGA) | 0.36 | 0.91 | 0.40 |
| Lys (AAG) | 0.36 | 1.01 | 0.35 |
| Thr (ACC) | 0.35 | 1.14 | 0.30 |
| Tyr (TAC) | 0.36 | 0.93 | 0.38 |
| pUA 552 | 0.35 | 1.01 | 0.35 |
| pMG2287 | 0.36 | 0.94 | 0.38 |

**Supplementary Equation 1 and 2**

- GS_m_ = Growth score of mistranslating strain;
- GS_c_ = Growth score of pUA552 control strain;
- A_id_ = Measured area for isolate “i” and spot dilution “d”;
- n_id_ = the set of all isolates in a given dilution for the strain under study;

$${GS}_{m}=\frac{1}{n_{id}}\sum_{nɛid} \left( \frac{\left[ A_{\mathrm{id}} \right]\mathrm{stress}}{\left[ A_{\mathrm{id}} \right]\mathrm{nostress}} \right)$$

Equation 1

$${GS}_{c}=\frac{1}{n_{id}}\sum_{nɛid} \left( \frac{\left[ A_{\mathrm{id}} \right]\mathrm{stress}}{\left[ A_{\mathrm{id}} \right]\mathrm{nostress}} \right)$$

Equation 2
