# Supplementary material for: The Fungus Candida albicans Tolerates Ambiguity at Multiple Codons
Source: Front Microbiol. 2016 Mar 31;7:401. doi: 10.3389/fmicb.2016.00401 (PMC4814463; doi:10.3389/fmicb.2016.00401)
Supplement: Supplementary file 7 [file Image1.PDF]

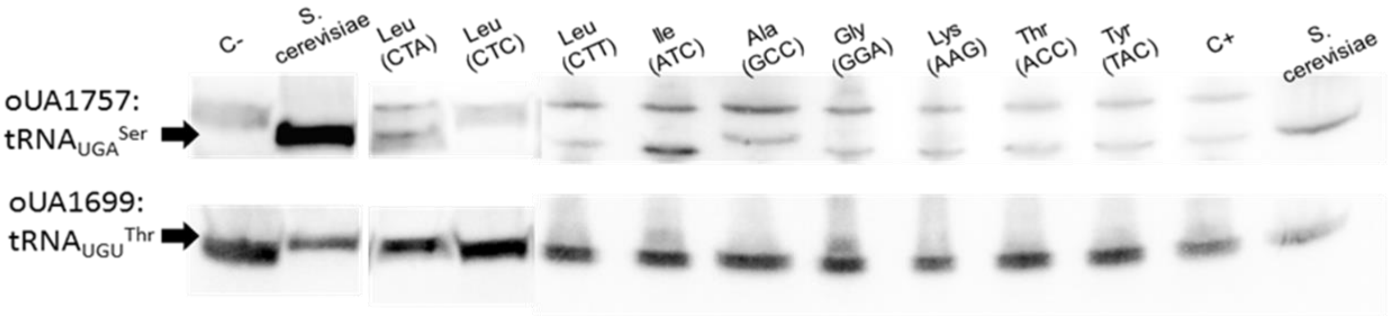

Supplementary Figure 1: Northern blot analysis of the mutant tRNAs. 50 µg of total RNA were resolved at room temperature on 15% polyacrylamide (40% Acril:Bis) gels containing 8 M urea. Probe oUA1757 labeled with ɣ-^{32}P-ATP was used to detect tRNA$_{UGA}^{Ser}$, probe oUA1699 labeled with ɣ-^{32}P-ATP was used to detect the internal control tRNA$_{UGU}^{Thr}$.
