# Supplementary material for: The Fungus Candida albicans Tolerates Ambiguity at Multiple Codons
Source: Front Microbiol. 2016 Mar 31;7:401. doi: 10.3389/fmicb.2016.00401 (PMC4814463; doi:10.3389/fmicb.2016.00401)
Supplement: Supplementary file 8 [file Image2.PDF]

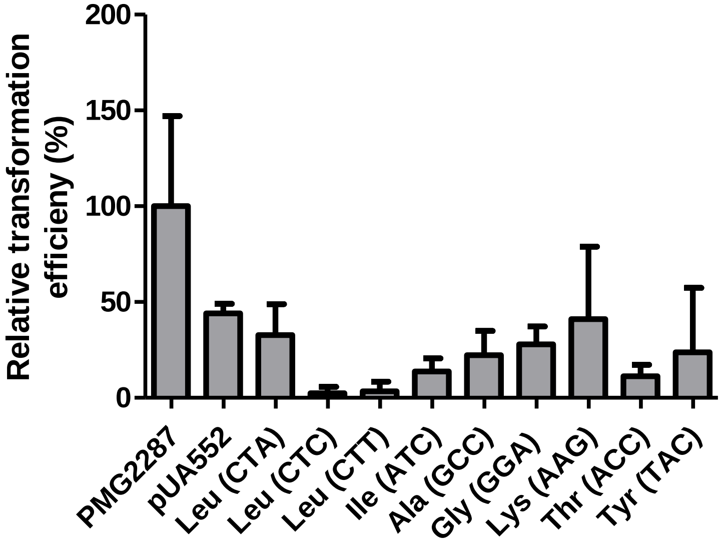

Supplementary Figure 2: Transformation efficiency. Transformation efficiency was determined using at least 3 independent transformations. Values obtained were normalized relative to the control PM2287 strain (%). Data are mean + s.d., and shows sharp reduction in the transformation efficiency of all misreading tRNA genes, relative to the control.
