# Supplementary material for: The Fungus Candida albicans Tolerates Ambiguity at Multiple Codons
Source: Front Microbiol. 2016 Mar 31;7:401. doi: 10.3389/fmicb.2016.00401 (PMC4814463; doi:10.3389/fmicb.2016.00401)
Supplement: Supplementary file 9 [file Image3.PDF]

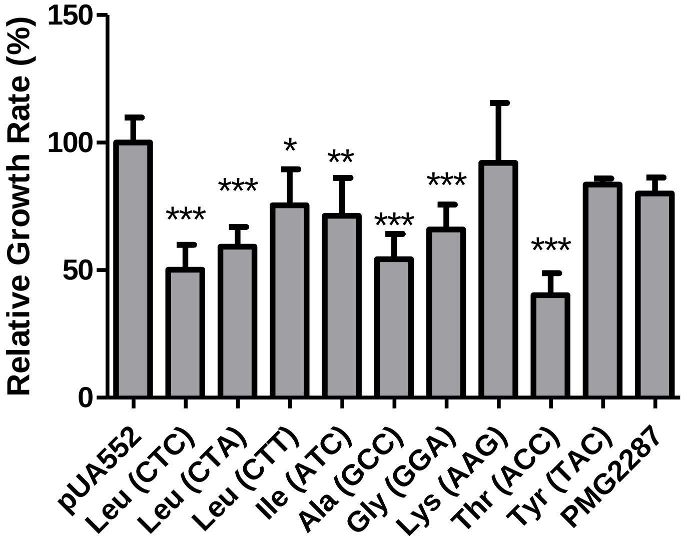

Supplementary Figure 3: Growth rate of mistranslating *C. albicans* strains. The data shows decreased growth rate in all strains, however strains misincorporating serine at chemically distinct sites show sharper negative effects. Data represents growth rate mean + s.d. of triplicates of 3 different clones, normalized to the pUA552 control. Data statistical analysis one-way ANOVA was performed followed by a Dunnet test with CI 95% relative to pUA552 (***$p<0.001$, **$p<0.01$, *$p<0.05$).
