# Supplementary material for: The Fungus Candida albicans Tolerates Ambiguity at Multiple Codons
Source: Front Microbiol. 2016 Mar 31;7:401. doi: 10.3389/fmicb.2016.00401 (PMC4814463; doi:10.3389/fmicb.2016.00401)
Supplement: Supplementary file 10 [file Image4.PDF]

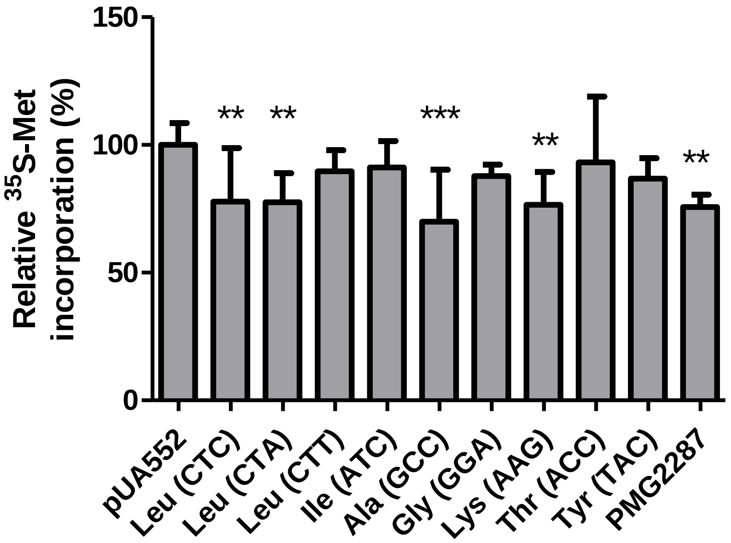

Supplementary Figure 4: Effect of mistranslation on protein synthesis rate. *C. albicans* cells were collected in exponential growth phase and incubated for 20 min in media lacking methionine. Cells were labelled with [^{35}S]-Met for 8 min and protein synthesis was then stopped by adding cycloheximide. Data represents mean incorporation of [^{35}S]-Met on new synthesized protein + s.d. of triplicates of 3 different clones, normalized with pUA552. Statistical analysis was carried out using one-way ANOVA followed by a Dunnet test with CI 95% relative to pUA552 (***$p<0.001$, **$p<0.01$, *$p<0.05$
