# Supplementary material for: The Fungus Candida albicans Tolerates Ambiguity at Multiple Codons
Source: Front Microbiol. 2016 Mar 31;7:401. doi: 10.3389/fmicb.2016.00401 (PMC4814463; doi:10.3389/fmicb.2016.00401)
Supplement: Supplementary file 11 [file Image5.PDF]

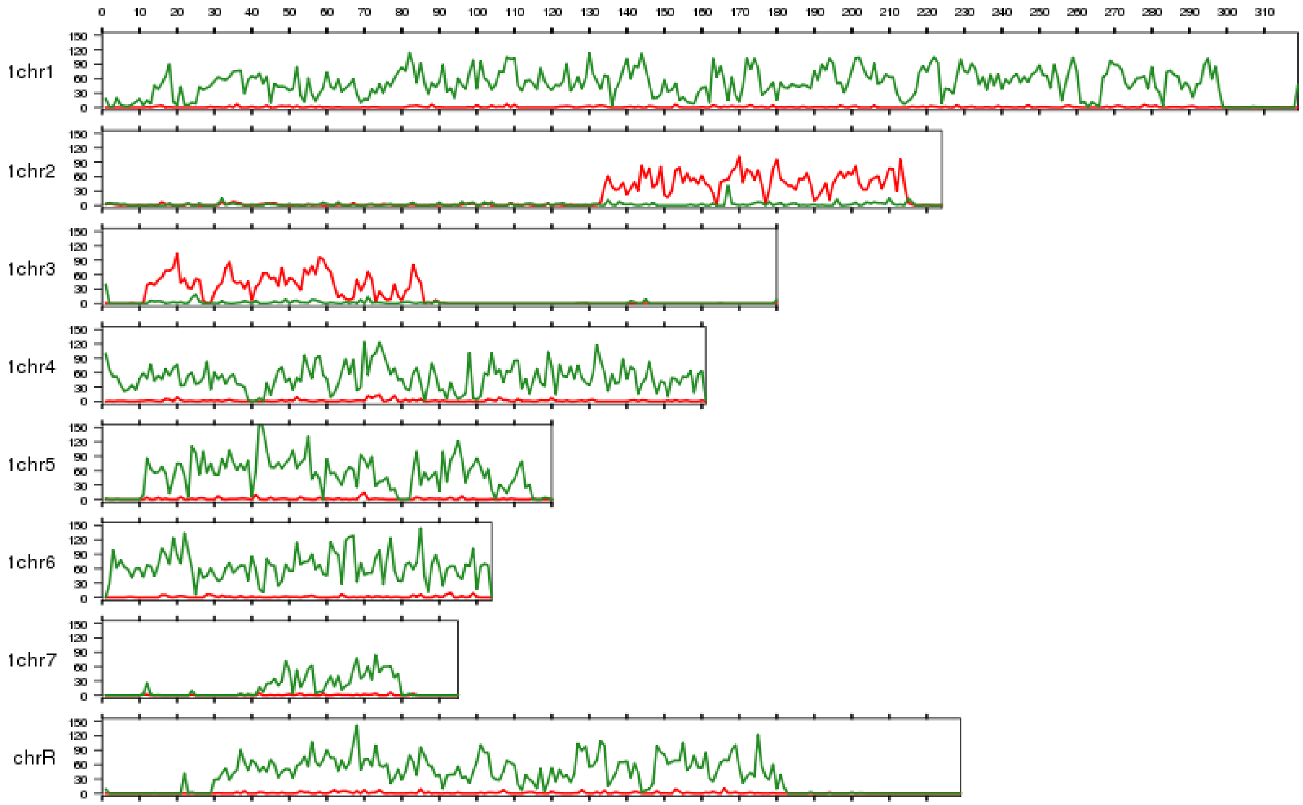

Supplementary Figure 5: Genomic analysis of the leu CTC (not evolved strain) and evolved strains without Amphotericin shows LOH in a region of chromosome 2 and 3 in leu(CTC). SNPs per kilobase are shaded green; density of LOHSNPs is in red, and gray vertical lines indicate the major repeat sequence.
