# Supplementary material for: The Fungus Candida albicans Tolerates Ambiguity at Multiple Codons
Source: Front Microbiol. 2016 Mar 31;7:401. doi: 10.3389/fmicb.2016.00401 (PMC4814463; doi:10.3389/fmicb.2016.00401)
Supplement: Supplementary file 12 [file Image6.PDF]

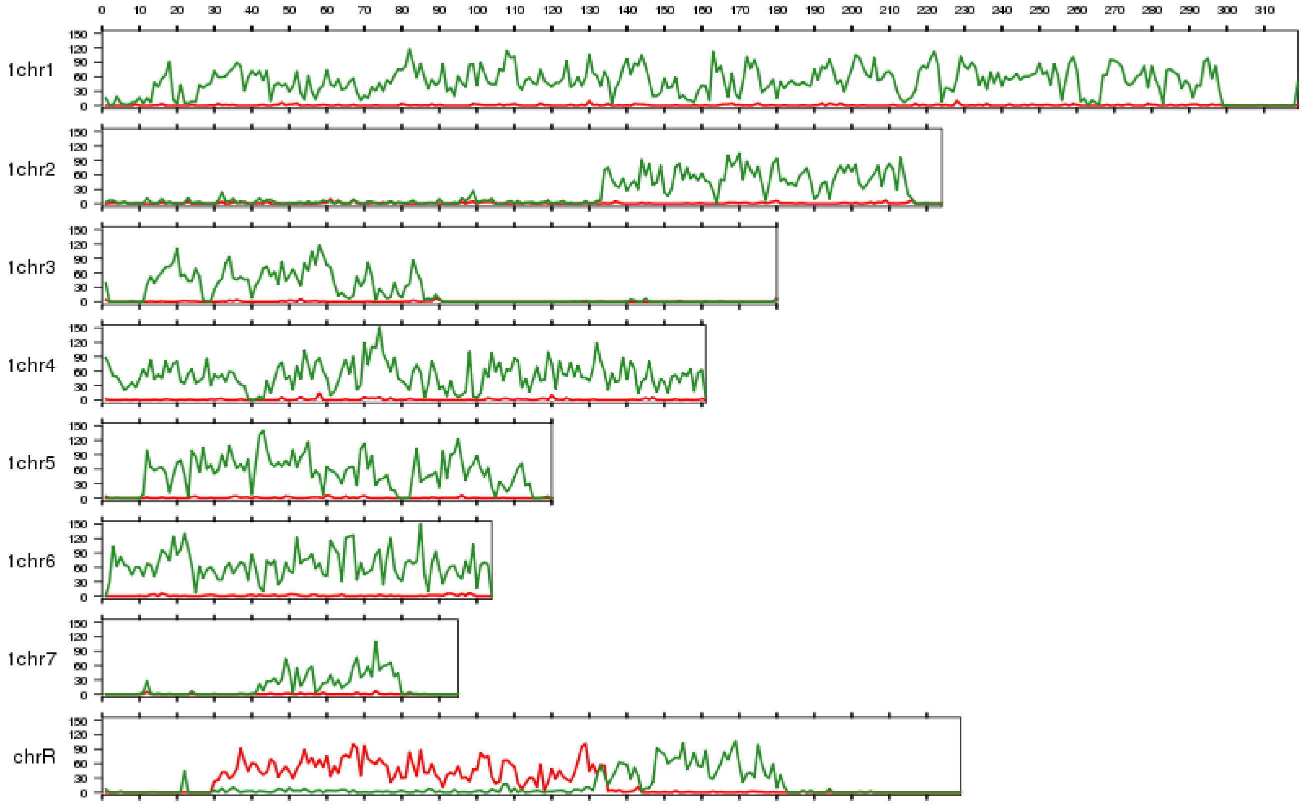


**Supplementary Figure 6:** Genomic analysis of the control (not evolved strain) and misincorporating strains shows LOH in a region of chromosome R in ala (GCC) . SNPs per kilobase are shaded green; density of LOHSNPs is in red, and gray vertical lines indicate the major repeat sequence.
