# Supplementary material for: The Fungus Candida albicans Tolerates Ambiguity at Multiple Codons
Source: Front Microbiol. 2016 Mar 31;7:401. doi: 10.3389/fmicb.2016.00401 (PMC4814463; doi:10.3389/fmicb.2016.00401)
Supplement: Supplementary file 13 [file Image7.PDF]

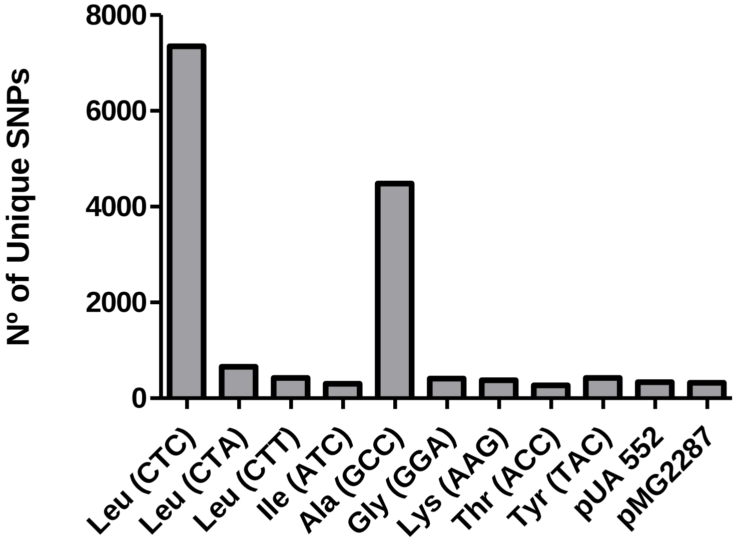

Supplementary Figure 7: Total number of unique SNP. Comparison between strains not evolved and respective evolved strains
